# The gut-inflammation-hearing axis in diabetic patients aged 30–55: exploring microbiome-mediated pathways to auditory complications

**DOI:** 10.1186/s12866-026-05504-8

**Published:** 2026-08-15

**Authors:** Peipei Zhang, Lili Jiang, Chaochao Ge, Jianlou Yang, Zhen Shi, Xiao Liang, Zhaobin Fan

**Affiliations:** 1 College of Pharmacy, Heze University, Heze, Shandong 274000 China; 2 Department of Health Services and Management, School of Sport Management, Shandong Sport University, Jinan, Shandong 250102 China

**Keywords:** Type 2 diabetes mellitus, Hearing impairment, Gut microbiota, Systemic inflammation, Lipopolysaccharide-binding protein, Mediation analysis, Biomarkers

## Abstract

**Background/Objectives:**

Hearing impairment is an underrecognized yet prevalent complication of type 2 diabetes mellitus (T2DM), but the underlying mechanisms remain poorly understood. This study investigated whether gut microbiota dysbiosis contributes to diabetic hearing loss through systemic inflammatory pathways, and evaluated the potential of microbiota-inflammation biomarkers for early risk identification.

**Methods:**

We conducted a cross-sectional study of 100 T2DM patients aged 30–55 years. Hearing function was assessed using pure-tone audiometry. Gut microbiota composition was analyzed by 16 S rRNA gene sequencing. Plasma inflammatory biomarkers including tumor necrosis factor-alpha (TNF-α), interleukin-6 (IL-6), interleukin-1β (IL-1β), high-sensitivity C-reactive protein (hs-CRP), and lipopolysaccharide-binding protein (LBP) were measured by ELISA. Mediation analyses with bootstrapping (5,000 resamples) examined whether inflammatory markers mediated the relationship between gut dysbiosis and hearing impairment. Receiver operating characteristic curves evaluated biomarker discriminatory ability.

**Results:**

Hearing impairment was present in 43% of participants. Patients with hearing impairment exhibited significant gut dysbiosis characterized by depletion of anti-inflammatory bacteria (*Faecalibacterium*: 6.2% vs. 9.8%, *P* < 0.001; *Roseburia*; *Akkermansia*) and enrichment of pro-inflammatory taxa (*Escherichia-Shigella*: 3.8% vs. 1.4%, *P* < 0.001; *Klebsiella*; *Desulfovibrio*). Inflammatory markers were significantly elevated, particularly LBP (18.6 vs. 12.4 µg/mL, *P* < 0.001), TNF-α (4.82 vs. 3.21 pg/mL, *P* < 0.001), and IL-6 (5.67 vs. 3.45 pg/mL, *P* < 0.001). Mediation analyses revealed that inflammatory pathways, predominantly through LBP-mediated gut barrier dysfunction, accounted for 48.7–56.3% of the total effect of microbiota alterations on hearing impairment (all *P* < 0.001). An integrated biomarker panel combining microbial taxa (*Faecalibacterium*, *Escherichia-Shigella*, Shannon diversity) and LBP achieved excellent discrimination for identifying hearing impairment (AUC = 0.921, 95% CI: 0.869–0.973; sensitivity 88.4%, specificity 87.7%).

**Conclusions:**

This study establishes a novel gut-inflammation-hearing axis in type 2 diabetes, wherein microbiota dysbiosis characterized by loss of beneficial bacteria and expansion of pathogenic taxa promotes hearing impairment through systemic inflammation mediated by intestinal barrier dysfunction and metabolic endotoxemia. These findings identify gut barrier integrity and associated inflammatory pathways as potential therapeutic targets, and demonstrate that integrated microbiota-inflammation biomarker panels can serve as non-invasive tools for early identification of hearing impairment risk in diabetic patients, enabling targeted preventive interventions.

**Supplementary Information:**

The online version contains supplementary material available at https://doi.org/10.1186/s12866-026-05504-8.

## Background

Type 2 diabetes mellitus (T2DM) has emerged as a global health crisis, affecting over 537 million adults worldwide, with projections estimating this number to reach 783 million by 2045 [1]. Beyond the well-established macrovascular and microvascular complications, accumulating evidence suggests that T2DM is associated with a significantly increased risk of hearing impairment, a often-overlooked complication that substantially impacts quality of life [2, 3]. Epidemiological studies have demonstrated that diabetic patients exhibit a 2.15-fold higher prevalence of hearing loss compared to non-diabetic individuals, with the risk escalating in middle-aged and older populations [4]. However, the underlying mechanisms linking diabetes to auditory dysfunction remain incompletely understood.

Recent advances in microbiome research have revolutionized our understanding of T2DM pathophysiology, revealing profound alterations in gut microbial composition and function in diabetic patients [5, 6]. The gut microbiota, comprising trillions of microorganisms, plays a crucial role in metabolic homeostasis through multiple pathways, including the regulation of glucose metabolism, production of short-chain fatty acids (SCFAs), and modulation of intestinal barrier integrity [7]. Dysbiosis in T2DM is characterized by reduced microbial diversity, decreased abundance of beneficial bacteria such as *Akkermansia muciniphila* and *Faecalibacterium prausnitzii*, and expansion of potentially pathogenic species [8, 9]. This microbial imbalance has been implicated in the pathogenesis of various diabetic complications through its influence on systemic inflammation and metabolic dysfunction [10].

The concept of chronic low-grade inflammation as a central driver of diabetic complications has gained substantial traction in recent years [11, 12]. Elevated circulating levels of pro-inflammatory cytokines, including tumor necrosis factor-alpha (TNF-α), interleukin-6 (IL-6), and C-reactive protein (CRP), are consistently observed in T2DM patients and correlate with disease progression and complication severity [13]. Emerging evidence suggests that gut dysbiosis contributes to systemic inflammation through multiple mechanisms: compromised intestinal barrier function leading to increased permeability (“leaky gut”), translocation of bacterial lipopolysaccharides (LPS) into systemic circulation, and altered production of anti-inflammatory metabolites [14, 15]. This gut-derived inflammation may represent a critical mechanistic link between metabolic dysregulation and end-organ damage.

The inner ear represents a highly metabolically active organ that is particularly vulnerable to inflammatory insults and microvascular damage [16]. The auditory system’s high energy demands and limited regenerative capacity make it susceptible to oxidative stress and inflammatory mediators [17]. Recent studies have begun to elucidate potential connections between systemic inflammation and cochlear dysfunction, with pro-inflammatory cytokines shown to induce apoptosis of cochlear hair cells and damage to the stria vascularis [18, 19]. Furthermore, microvascular complications in diabetes, driven in part by inflammatory processes, may compromise cochlear blood supply and oxygen delivery, leading to progressive hearing deterioration [20].

Despite these individual lines of evidence, the potential interaction between gut microbiota dysbiosis, systemic inflammation, and hearing impairment in diabetic patients remains unexplored. We hypothesize the existence of a “gut-inflammation-hearing axis” wherein diabetes-associated gut dysbiosis promotes systemic inflammation, which subsequently contributes to cochlear damage and hearing loss. Understanding this tripartite relationship could unveil novel therapeutic targets for preventing or mitigating diabetic auditory complications.

The present study aims to investigate the associations among gut microbiota composition, circulating inflammatory biomarkers, and hearing function in middle-aged and older adults (aged 30–55 years) with T2DM. Specifically, we seek to: (1) characterize the gut microbial profiles of diabetic patients with and without hearing impairment; (2) examine the relationship between gut dysbiosis and systemic inflammatory markers; (3) identify specific microbial taxa and inflammatory mediators associated with auditory dysfunction; and (4) explore potential microbiome-mediated inflammatory pathways linking diabetes to hearing complications. By elucidating these interconnections, this research may pave the way for microbiome-targeted interventions to preserve hearing function in the growing population of individuals with diabetes.

## Methods

### Study design and participants

This cross-sectional study was conducted between January 2024 and December 2024 at Heze Municipal Hospital. A total of 100 participants aged 30–55 years with diagnosed type 2 diabetes mellitus were recruited from the outpatient endocrinology clinic. The diagnosis of T2DM was established according to the American Diabetes Association criteria: fasting plasma glucose ≥ 7.0 mmol/L, 2-hour plasma glucose ≥ 11.1 mmol/L during oral glucose tolerance test, or glycated hemoglobin (HbA1c) ≥ 6.5% [21].

Inclusion criteria were: (1) age 30–55 years; (2) confirmed diagnosis of T2DM for at least 6 months; (3) clinically stable condition without acute complications; and (4) willingness to provide written informed consent. Exclusion criteria included: (1) type 1 diabetes or secondary diabetes; (2) antibiotic use within 3 months prior to enrollment; (3) probiotic or prebiotic supplementation within 1 month; (4) history of gastrointestinal surgery or inflammatory bowel disease; (5) chronic ear diseases or previous ear surgery; (6) occupational noise exposure or ototoxic medication use; (7) pregnancy or lactation; (8) malignancy or severe organ dysfunction; and (9) immunosuppressive therapy or corticosteroid use.

The study protocol was approved by the Institutional Review Ethics Committee of Heze Municipal Hospital (Approval No: 2023-KY017) and was conducted in accordance with the Declaration of Helsinki. All participants provided written informed consent prior to enrollment.

### Clinical and anthropometric assessments

Comprehensive clinical data were collected for all participants, including age, sex, body mass index (BMI), duration of diabetes, smoking status, alcohol consumption, and medication history. Blood pressure was measured in triplicate after 5 min of rest using a standard sphygmomanometer, and the average of three readings was recorded. Fasting plasma glucose, HbA1c, lipid profiles (total cholesterol, triglycerides, low-density lipoprotein cholesterol, high-density lipoprotein cholesterol), and renal function parameters (serum creatinine, estimated glomerular filtration rate) were assessed using standard laboratory methods [22].

### Hearing function assessment

Hearing function was evaluated by certified audiologists blinded to the participants’ clinical and laboratory data. Pure-tone audiometry was performed in a soundproof booth using a clinical audiometer (model [1066], GN Otometrics) calibrated according to ISO standards [23]. Air conduction thresholds were measured at frequencies of 0.25, 0.5, 1, 2, 4, and 8 kHz for both ears. The pure-tone average (PTA) was calculated as the mean threshold at 0.5, 1, 2, and 4 kHz [24].

Hearing impairment was defined as PTA > 25 dB HL in the better-hearing ear, consistent with World Health Organization criteria [25]. The severity of hearing loss was classified as mild (26–40 dB HL), moderate (41–60 dB HL), or severe (> 60 dB HL). Tympanometry was performed to exclude middle ear pathology that could confound results [26].

### Fecal sample collection and gut microbiota analysis

Participants were provided with sterile fecal collection kits and detailed instructions for home collection. Fresh stool samples were collected in the morning and immediately stored at -20 °C in home freezers, then transported to the laboratory on dry ice within 24 h and stored at -80 °C until analysis [27].

Microbial DNA was extracted from approximately 200 mg of fecal sample using the QIAamp PowerFecal DNA Kit (Qiagen, Germany) according to the manufacturer’s protocol. DNA concentration and purity were assessed using a NanoDrop spectrophotometer (Thermo Fisher Scientific) and agarose gel electrophoresis [28].

The V3-V4 hypervariable regions of the bacterial 16 S rRNA gene were amplified using universal primers 338 F (5’-ACTCCTACGGGAGGCAGCAG-3’) and 806R (5’-GGACTACHVGGGTWTCTAAT-3’) [29]. PCR amplification was performed in 25 µL reaction volumes containing 12.5 µL of 2× Phusion High-Fidelity PCR Master Mix, 2.5 µL of each primer (5 µM), 2 µL of template DNA, and 5.5 µL of nuclease-free water. The thermal cycling conditions were: initial denaturation at 98 °C for 30 s; 30 cycles of denaturation at 98 °C for 10 s, annealing at 54 °C for 30 s, and extension at 72 °C for 45 s; and final extension at 72 °C for 10 min [30].

PCR products were purified using AMPure XP beads (Beckman Coulter) and quantified using a Qubit fluorometer (Thermo Fisher Scientific). Equimolar amounts of purified amplicons were pooled and sequenced on an Illumina NovaSeq 6000 platform with 2 × 250 bp paired-end reads (Illumina, San Diego, CA, USA) [31].

### Microbiota bioinformatics analysis

Raw sequencing reads were processed using QIIME 2 (version 2023.5) pipeline [32]. Briefly, paired-end reads were quality-filtered, denoised, and merged using DADA2 algorithm to generate amplicon sequence variants (ASVs) [33]. Chimeric sequences were identified and removed using the consensus method. Taxonomic assignment was performed against the SILVA 138 reference database using a naive Bayes classifier with 99% similarity threshold [34].

Alpha diversity indices, including observed ASVs, Shannon index, Simpson index, and Chao1 richness estimator, were calculated to assess within-sample microbial diversity [35]. Beta diversity was evaluated using weighted and unweighted UniFrac distances and visualized through principal coordinates analysis (PCoA) [36]. Differential abundance analysis of bacterial taxa between groups was conducted using Linear discriminant analysis Effect Size (LEfSe) with a logarithmic LDA score threshold of 2.0 [37].

### Blood sample collection and inflammatory biomarker measurement

Venous blood samples (5 mL) were collected after overnight fasting (≥ 8 h) using EDTA-coated tubes. Samples were centrifuged at 3,000 rpm for 15 min at 4 °C within 2 h of collection, and plasma was aliquoted and stored at -80 °C until analysis [38].

Plasma concentrations of inflammatory biomarkers were measured using commercially available enzyme-linked immunosorbent assay (ELISA) kits according to manufacturers’ protocols. The following cytokines were quantified: tumor necrosis factor-alpha (TNF-α), interleukin-6 (IL-6), interleukin-1β (IL-1β), interleukin-10 (IL-10), and high-sensitivity C-reactive protein (hs-CRP) (R&D Systems, Minneapolis, MN, USA) [39]. Lipopolysaccharide-binding protein (LBP), a marker of bacterial translocation and gut barrier dysfunction, was also measured using ELISA (Hycult Biotech, The Netherlands) [40]. All samples were analyzed in duplicate, and the intra-assay and inter-assay coefficients of variation were < 10% and < 15%, respectively.

### Statistical analysis

Statistical analyses were performed using R software (version 4.3.0) and SPSS (version 26.0, IBM Corp., Armonk, NY, USA). Continuous variables were expressed as mean ± standard deviation (SD) or median with interquartile range (IQR) depending on the distribution. Categorical variables were presented as frequencies and percentages. Normality was assessed using the Shapiro-Wilk test and visual inspection of Q-Q plots [41].

Comparisons between participants with and without hearing impairment were conducted using independent t-tests for normally distributed continuous variables, Mann-Whitney U tests for non-normally distributed variables, and chi-square tests or Fisher’s exact tests for categorical variables [42].

Spearman’s rank correlation coefficients were calculated to examine associations between gut microbiota composition (relative abundance of bacterial taxa), inflammatory biomarkers, and hearing thresholds. Partial correlation analyses were performed adjusting for potential confounders including age, sex, BMI, diabetes duration, and HbA1c levels [43].

Multiple linear regression models were constructed to identify independent predictors of hearing threshold, with covariates selected based on biological plausibility and univariate associations (*P* < 0.10). Multicollinearity was assessed using variance inflation factors (VIF < 5 considered acceptable) [44].

Mediation analysis was performed using the bootstrapping method with 5,000 resamples to examine whether inflammatory biomarkers mediate the relationship between gut microbiota composition and hearing impairment, using the PROCESS macro for SPSS [45]. The significance of indirect effects was determined by 95% bias-corrected confidence intervals not crossing zero.

A two-sided P-value < 0.05 was considered statistically significant for all analyses. False discovery rate (FDR) correction using the Benjamini-Hochberg method was applied for multiple comparisons in microbiota differential abundance analyses [46].

Sample size was calculated based on an anticipated correlation coefficient of 0.30 between gut microbiota diversity and inflammatory markers, with 80% power and a two-sided significance level of 0.05. This yielded a minimum required sample size of 84 participants. To account for potential dropouts and incomplete data, we enrolled 100 participants [47].

## Results

### Participant characteristics and hearing status

A total of 100 middle-aged and older adults with type 2 diabetes mellitus (T2DM) were enrolled in this study. The cohort comprised 58 males and 42 females, with a mean age of 45.3 ± 6.8 years (range: 30–55 years). The average duration of diabetes was 7.2 ± 4.5 years, and the mean HbA1c level was 7.8 ± 1.4%, indicating suboptimal glycemic control in a substantial proportion of participants. The mean body mass index (BMI) was 26.4 ± 3.7 kg/m², with 62% of participants classified as overweight or obese (BMI ≥ 25 kg/m²).

Based on pure-tone audiometry assessment, 43 participants (43.0%) were identified as having hearing impairment (PTA > 25 dB HL in the better-hearing ear), while 57 participants (57.0%) had normal hearing. Among those with hearing impairment, 28 (65.1%) had mild hearing loss (26–40 dB HL), 13 (30.2%) had moderate hearing loss (41–60 dB HL), and 2 (4.7%) had severe hearing loss (> 60 dB HL). The mean PTA was significantly higher in the hearing impairment group compared to the normal hearing group (34.8 ± 8.6 dB HL vs. 18.2 ± 4.3 dB HL, *P* < 0.001). High-frequency hearing loss (at 4 and 8 kHz) was more prevalent than low-frequency hearing loss, a pattern that has been reported in metabolic-related auditory dysfunction. Notably, this high-frequency-predominant pattern is also characteristic of age-related presbycusis, and given the substantial age difference between groups (see below), the present cross-sectional design cannot fully distinguish age-related from diabetes- or inflammation-related contributions to the hearing loss observed.

Comparative analysis of baseline characteristics between participants with and without hearing impairment revealed several significant differences (Table 1). Participants with hearing impairment were significantly older (48.7 ± 5.9 years vs. 42.7 ± 6.4 years, *P* < 0.001) and had longer diabetes duration (9.5 ± 4.8 years vs. 5.5 ± 3.4 years, *P* < 0.001) compared to those with normal hearing. Moreover, the hearing impairment group exhibited poorer glycemic control, as evidenced by higher HbA1c levels (8.4 ± 1.5% vs. 7.4 ± 1.2%, *P* = 0.001) and fasting plasma glucose concentrations (8.9 ± 2.3 mmol/L vs. 7.6 ± 1.8 mmol/L, *P* = 0.002).

**Table 1:** Baseline characteristics of study participants

Characteristic	Total (*n* = 100)	Normal Hearing (*n* = 57)	Hearing Impairment (*n* = 43)	*P*-value
Demographics				
Age(years)	45.3 ± 6.8	42.7 ± 6.4	48.7 ± 5.9	< 0.001***
Male sex, n(%)	58(58.0)	35(61.4)	23(53.5)	0.482
BMI(kg/m²)	26.4 ± 3.7	26.1 ± 3.5	26.9 ± 4.0	0.256
Current smoker, n(%)	28(28.0)	18(31.6)	10(23.3)	0.391
Alcohol consumption, n (%)	32(32.0)	20(35.1)	12(27.9)	0.564
Diabetes-related parameters				
Diabetes duration(years)	7.2 ± 4.5	5.5 ± 3.4	9.5 ± 4.8	< 0.001***
HbA1c(%)	7.8 ± 1.4	7.4 ± 1.2	8.4 ± 1.5	0.001**
FPG(mmol/L)	8.2 ± 2.1	7.6 ± 1.8	8.9 ± 2.3	0.002**
Cardiovascular parameters				
SBP(mmHg)	128.6 ± 14.5	127.2 ± 13.8	130.4 ± 15.3	0.183
DBP(mmHg)	81.3 ± 9.7	80.6 ± 9.2	82.2 ± 10.4	0.421
Total cholesterol(mmol/L)	5.1 ± 1.0	5.0 ± 0.9	5.2 ± 1.1	0.312
Triglycerides(mmol/L)	2.0 ± 1.1	1.9 ± 1.0	2.1 ± 1.2	0.428
LDL-C(mmol/L)	3.1 ± 0.8	3.0 ± 0.7	3.2 ± 0.9	0.267
HDL-C(mmol/L)	1.2 ± 0.3	1.2 ± 0.3	1.2 ± 0.3	0.531
Renal function				
Serum creatinine(µmol/L)	79.3 ± 17.2	76.8 ± 15.2	82.4 ± 18.6	0.089
eGFR(mL/min/1.73 m²)	91.8 ± 15.8	94.6 ± 14.3	88.3 ± 16.7	0.045*
Hearing assessment				
PTA at 0.5–4 kHz(dBHL)	25.4 ± 10.2	18.2 ± 4.3	34.8 ± 8.6	< 0.001***
Threshold at 4 kHz(dB HL)	32.8 ± 15.6	22.1 ± 8.7	46.7 ± 13.2	< 0.001***
Threshold at 8 kHz(dB HL)	38.5 ± 18.3	26.4 ± 10.5	54.2 ± 16.8	< 0.001***
Medication use, n(%)				
Metformin	79(79.0)	46(80.7)	33(76.7)	0.619
Sulfonylureas	30(30.0)	16(28.1)	14(32.6)	0.621
DPP-4 inhibitors	37(37.0)	20(35.1)	17(39.5)	0.654
Insulin	16(16.0)	8(14.0)	8(18.6)	0.532
Statins	51(51.0)	28(49.1)	23(53.5)	0.661
Antihypertensive agents	44(44.0)	24(42.1)	20(46.5)	0.658

Data are presented as mean ± SD or n (%). *BMI* Body mass index, *HbA1c* Glycated hemoglobin, *FPG* Fasting plasma glucose, *SBP* Systolic blood pressure, *DBP* Diastolic blood pressure, *LDL-C* Low-density lipoprotein cholesterol, *HDL-C* High-density lipoprotein cholesterol, *eGFR* Estimated glomerular filtration rate, *PTA* Pure-tone average, *DPP-4* Dipeptidyl peptidase-4

*P*-values were determined using independent t-tests for continuous variables and chi-square tests for categorical variables

*P < 0.05, **P < 0.01, ***P < 0.001

No significant differences were observed between the two groups in terms of sex distribution (*P* = 0.482), BMI (*P* = 0.256), smoking status (*P* = 0.391), or alcohol consumption (*P* = 0.564). Blood pressure measurements, including systolic and diastolic blood pressure, were comparable between groups (*P* = 0.183 and *P* = 0.421, respectively). Lipid profiles showed no significant differences in total cholesterol (*P* = 0.312), triglycerides (*P* = 0.428), LDL-cholesterol (*P* = 0.267), or HDL-cholesterol (*P* = 0.531) between participants with and without hearing impairment.

Regarding renal function, participants with hearing impairment demonstrated a trend toward higher serum creatinine levels (82.4 ± 18.6 µmol/L vs. 76.8 ± 15.2 µmol/L, *P* = 0.089) and lower estimated glomerular filtration rate (eGFR) (88.3 ± 16.7 mL/min/1.73 m² vs. 94.6 ± 14.3 mL/min/1.73 m², *P* = 0.045), suggesting a potential association between diabetic microvascular complications affecting both the kidney and cochlea. However, no participants met criteria for chronic kidney disease (eGFR < 60 mL/min/1.73 m²).

Medication use patterns were similar between groups, with comparable proportions receiving metformin (76.7% vs. 80.7%, *P* = 0.619), sulfonylureas (32.6% vs. 28.1%, *P* = 0.621), DPP-4 inhibitors (39.5% vs. 35.1%, *P* = 0.654), and insulin therapy (18.6% vs. 14.0%, *P* = 0.532). Statin use was also comparable (53.5% vs. 49.1%, *P* = 0.661), as was antihypertensive medication use (46.5% vs. 42.1%, *P* = 0.658).

These findings indicate that hearing impairment in our T2DM cohort is associated with older age, longer disease duration, and poorer glycemic control, but not with traditional cardiovascular risk factors such as lipid abnormalities or blood pressure elevation. The high prevalence (43%) of hearing impairment in this middle-aged diabetic population, while likely influenced by the clinical characteristics of our outpatient endocrinology cohort (including longer disease duration and poorer glycemic control), underscores the clinical significance of this often-overlooked complication and supports the rationale for investigating underlying mechanisms involving gut microbiota and systemic inflammation.

### Gut microbiota composition and diversity in diabetic patients with hearing impairment

#### Alpha diversity analysis

**Fig. 1:**
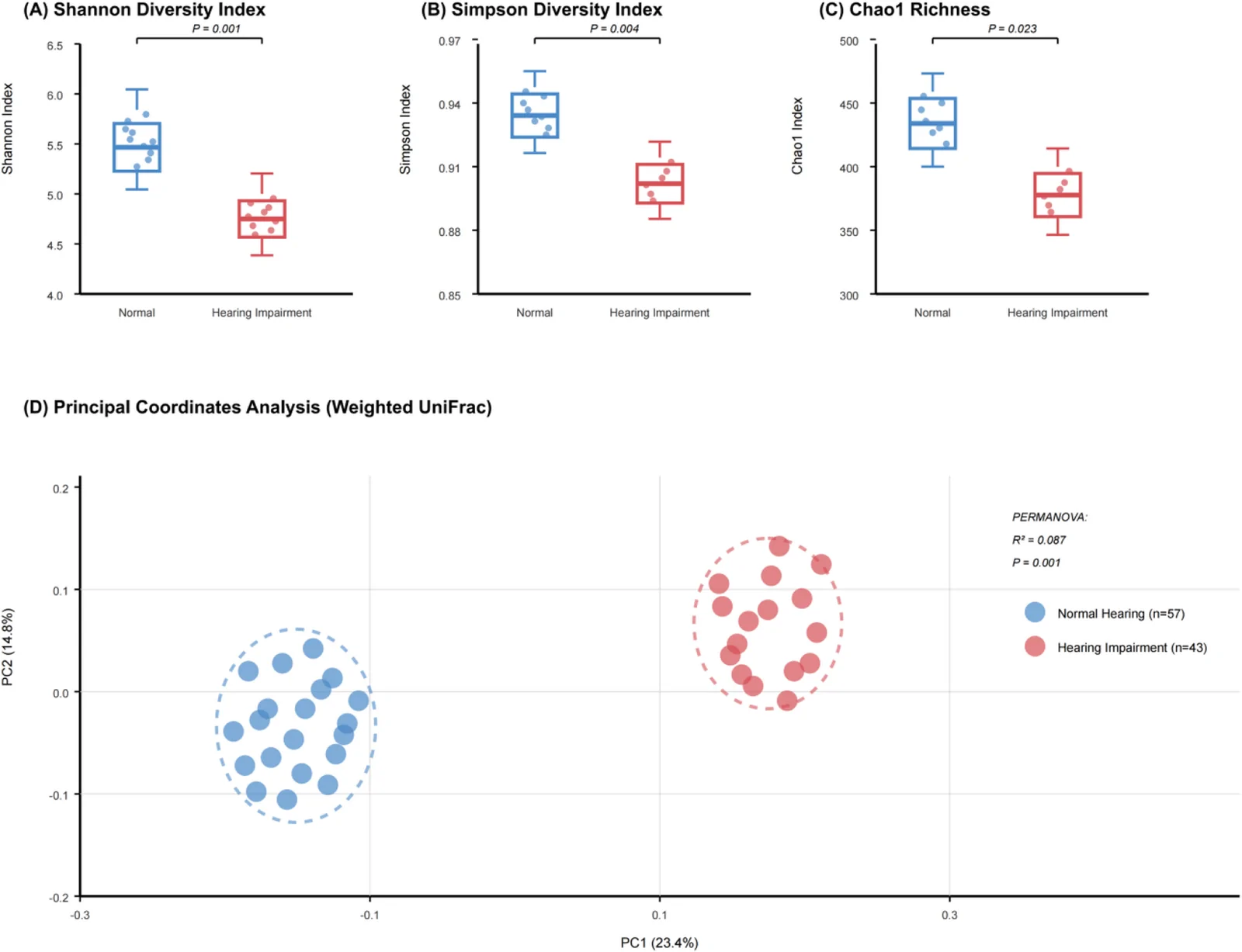
Gut microbiota diversity and composition in diabetic patients with and without hearing impairment. **A**-**C** Alpha diversity indices showing reduced microbial diversity in hearing impairment group. **D** Principal coordinates analysis (PCoA) based on weighted UniFrac distances demonstrating distinct clustering patterns between groups. Box plots show median, interquartile range, and individual data points

Analysis of within-sample microbial diversity revealed significant alterations in the gut microbiota of diabetic patients with hearing impairment compared to those with normal hearing (Fig. 1A-C). The Shannon diversity index, which accounts for both richness and evenness of microbial communities, was significantly reduced in the hearing impairment group (5.32 ± 0.68) compared to the normal hearing group (5.78 ± 0.54, *P* = 0.001), indicating lower overall microbial diversity. Similarly, the Simpson index demonstrated significantly decreased diversity in participants with hearing impairment (0.921 ± 0.045 vs. 0.946 ± 0.032, *P* = 0.004). The Chao1 richness estimator, which estimates the total number of species present, showed a trend toward lower species richness in the hearing impairment group (428.6 ± 89.3 vs. 467.2 ± 76.5, *P* = 0.023), though the magnitude of difference was less pronounced than diversity indices. The number of observed amplicon sequence variants (ASVs) was also significantly lower in patients with hearing impairment (394.8 ± 85.2 vs. 438.7 ± 71.6, *P* = 0.008).

These findings suggest that hearing impairment in diabetic patients is associated with reduced gut microbial diversity and richness, consistent with the concept of dysbiosis. After adjusting for potential confounders including age, diabetes duration, and HbA1c levels using partial correlation analysis, the association between Shannon diversity and hearing impairment remained significant (*r* = -0.312, *P* = 0.002), indicating that the observed microbial alterations are independently associated with auditory dysfunction beyond the effects of metabolic control and disease duration. The reduction in alpha diversity is particularly noteworthy, as decreased microbial diversity has been linked to impaired intestinal barrier function, enhanced systemic inflammation, and increased susceptibility to metabolic complications in previous studies of diabetes and other chronic diseases.

### Beta diversity and microbial community structure

Principal coordinates analysis (PCoA) based on weighted UniFrac distances revealed distinct clustering patterns between diabetic patients with and without hearing impairment, indicating significant differences in overall microbial community composition (Fig. 1D). The two groups showed clear separation along the first principal coordinate axis (PC1), which explained 23.4% of the total variance in microbial community structure. PERMANOVA analysis confirmed that hearing status was significantly associated with gut microbiota composition (R² = 0.087, *P* = 0.001, 999 permutations), indicating that approximately 8.7% of the variance in microbial community structure could be attributed to differences in hearing function. When using unweighted UniFrac distances, which give equal weight to all bacterial taxa regardless of their abundance, the separation between groups remained significant but was less pronounced (R² = 0.065, *P* = 0.004), suggesting that both abundant and rare taxa contribute to the compositional differences, with more abundant taxa playing a predominant role.

To account for potential confounding effects of demographic and clinical variables, we performed PERMANOVA with adjustment for age, sex, BMI, diabetes duration, and HbA1c levels. Even after these adjustments, hearing status remained a significant determinant of microbial community structure (adjusted R² = 0.061, *P* = 0.006), confirming that the observed compositional differences are not merely reflections of differences in age or metabolic control between groups. Furthermore, analysis of dispersion using the betadisper function revealed that the hearing impairment group exhibited greater within-group variability in microbial composition compared to the normal hearing group (*P* = 0.038), suggesting increased inter-individual heterogeneity in gut microbiota among diabetic patients with auditory dysfunction. This increased variability may reflect diverse pathogenic mechanisms or different stages of dysbiosis progression in the hearing impairment population, warranting further investigation of subgroups within this cohort.

### Differential abundance of bacterial taxa

**Fig. 2:**
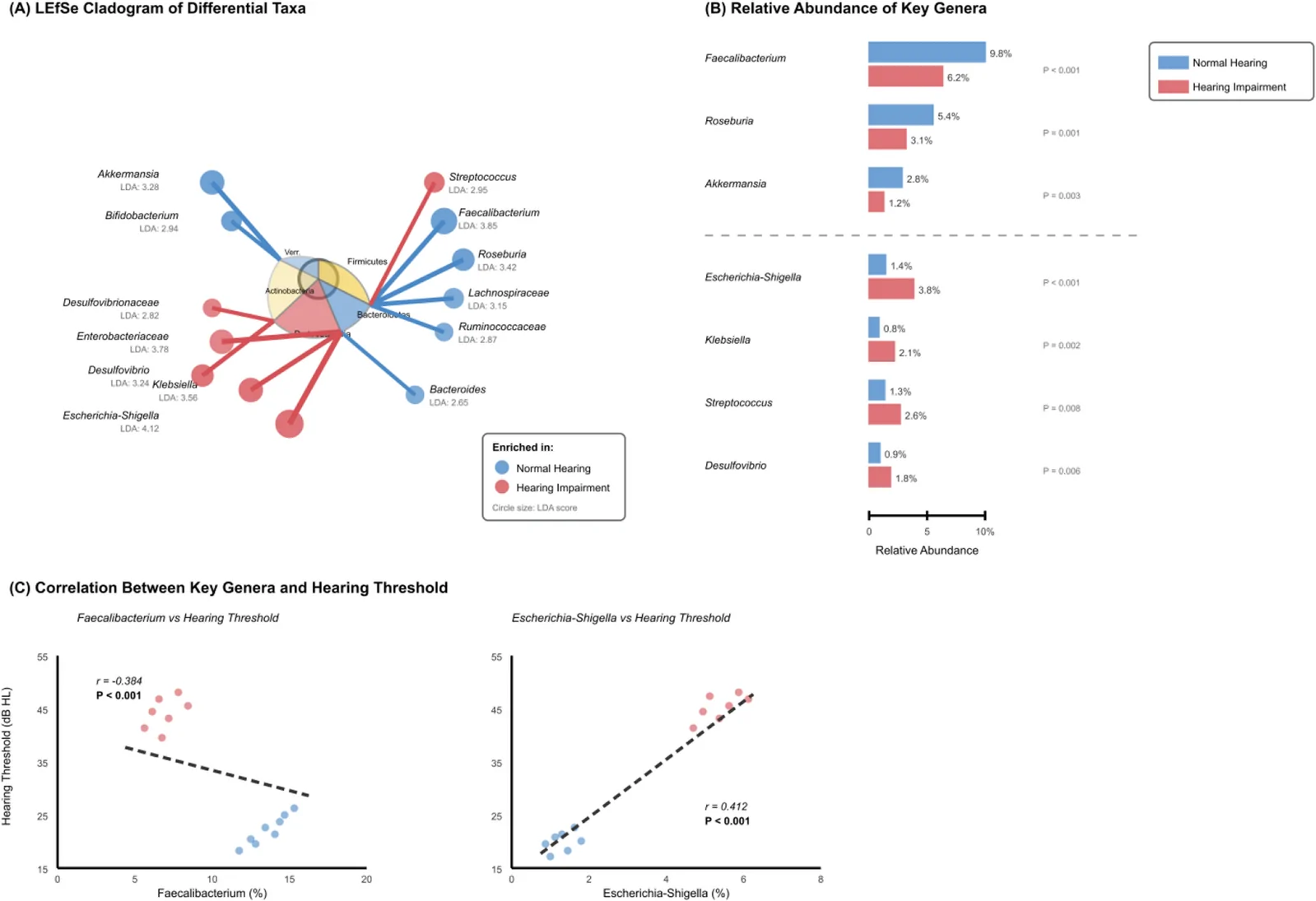
Differential abundance of gut bacterial taxa in diabetic patients with hearing impairment. **A** LEfSe cladogram showing discriminative bacterial taxa (LDA score > 2.0). Circle size represents LDA effect size. Blue: enriched in normal hearing; Red: enriched in hearing impairment. **B** Relative abundance of key genera with significant differences between groups. **C** Correlation analysis between representative genera and hearing threshold at 0.5–4 kHz. Blue dots: normal hearing; Red dots: hearing impairment. Dashed line: linear regression. All P-values determined by Mann-Whitney U test with FDR correction

Linear discriminant analysis effect size (LEfSe) analysis identified 28 bacterial taxa that were significantly differentially abundant between diabetic patients with and without hearing impairment (LDA score > 2.0, *P* < 0.05) (Fig. 2A). At the phylum level, the hearing impairment group showed a significantly higher relative abundance of Proteobacteria (18.3% vs. 12.6%, *P* = 0.003) and a lower abundance of Bacteroidetes (32.4% vs. 38.7%, *P* = 0.008) compared to the normal hearing group. The Firmicutes to Bacteroidetes (F/B) ratio was significantly elevated in participants with hearing impairment (1.68 vs. 1.42, *P* = 0.012), a pattern previously associated with metabolic dysfunction and increased intestinal permeability.

At the family level, several taxonomic groups demonstrated notable differences. The hearing impairment group exhibited significantly decreased abundances of beneficial bacterial families including Lachnospiraceae (12.8% vs. 16.3%, *P* = 0.002) and Ruminococcaceae (8.4% vs. 11.7%, *P* = 0.004), both of which are major producers of short-chain fatty acids (SCFAs) with anti-inflammatory properties. Conversely, potentially pathogenic families were enriched in the hearing impairment group, including Enterobacteriaceae (4.6% vs. 2.1%, *P* < 0.001) and Desulfovibrionaceae (1.8% vs. 0.9%, *P* = 0.006), which are associated with endotoxin production and intestinal inflammation.

At the genus level, the most striking differences were observed for several key taxa with established roles in metabolic health and immune regulation (Fig. 2B). The hearing impairment group showed significantly reduced relative abundances of *Faecalibacterium* (6.2% vs. 9.8%, *P* < 0.001), *Roseburia* (3.1% vs. 5.4%, *P* = 0.001), and *Akkermansia* (1.2% vs. 2.8%, *P* = 0.003). *Faecalibacterium prausnitzii*, the predominant species within the *Faecalibacterium* genus, is a major butyrate producer with potent anti-inflammatory properties and has been consistently depleted in various inflammatory and metabolic disorders. *Roseburia* species are also important butyrate producers that maintain intestinal barrier integrity. *Akkermansia muciniphila*, a mucin-degrading bacterium residing in the mucus layer, has been shown to improve metabolic health and reduce inflammation in diabetes.

In contrast, several genera were significantly enriched in the hearing impairment group, including *Escherichia-Shigella* (3.8% vs. 1.4%, *P* < 0.001), *Klebsiella* (2.1% vs. 0.8%, *P* = 0.002), *Streptococcus* (2.6% vs. 1.3%, *P* = 0.008), and *Desulfovibrio* (1.8% vs. 0.9%, *P* = 0.006). The *Escherichia-Shigella* group, which includes *Escherichia coli*, is a major source of lipopolysaccharide (LPS), a potent immunostimulatory molecule that can trigger systemic inflammation when translocated across a compromised intestinal barrier. *Klebsiella* species have been implicated in autoimmune conditions and metabolic endotoxemia. *Desulfovibrio* produces hydrogen sulfide, which at elevated levels can damage the intestinal epithelium and promote inflammation.

Correlation analysis between differential genera and clinical parameters revealed that the relative abundance of *Faecalibacterium* was negatively correlated with diabetes duration (*r* = -0.342, *P* = 0.001), HbA1c levels (*r* = -0.298, *P* = 0.003), and hearing threshold at 4 kHz (*r* = -0.384, *P* < 0.001), suggesting that depletion of this beneficial genus is associated with both poor glycemic control and auditory dysfunction. Conversely, *Escherichia-Shigella* abundance was positively correlated with HbA1c (*r* = 0.326, *P* = 0.001) and hearing threshold (*r* = 0.412, *P* < 0.001). These correlations remained significant after adjusting for age, sex, and BMI in partial correlation analyses, indicating independent associations between specific microbial taxa and hearing impairment in diabetes.

The observed pattern of microbial dysbiosis—characterized by depletion of SCFA-producing, anti-inflammatory bacteria and enrichment of pro-inflammatory, LPS-producing taxa—suggests a mechanistic link between gut microbiota alterations and hearing impairment through inflammation-mediated pathways. These findings provide a foundation for investigating the potential role of inflammatory biomarkers as mediators of the microbiota-hearing relationship, which is explored in subsequent analyses.

### Systemic inflammatory biomarkers and their association with hearing function

Comparative analysis of plasma inflammatory biomarkers revealed significant elevations in multiple pro-inflammatory mediators in diabetic patients with hearing impairment compared to those with normal hearing (Table 2). Tumor necrosis factor-alpha (TNF-α), a key pro-inflammatory cytokine involved in metabolic dysfunction and tissue damage, was significantly higher in the hearing impairment group (4.82 ± 1.36 pg/mL vs. 3.21 ± 0.98 pg/mL, *P* < 0.001). Similarly, interleukin-6 (IL-6), which plays a central role in acute-phase inflammatory responses and has been implicated in cochlear damage, showed markedly elevated levels in participants with hearing impairment (5.67 ± 1.84 pg/mL vs. 3.45 ± 1.12 pg/mL, *P* < 0.001). Interleukin-1β (IL-1β), another potent pro-inflammatory cytokine, was also significantly increased in the hearing impairment group (2.85 ± 0.94 pg/mL vs. 1.92 ± 0.68 pg/mL, *P* < 0.001). High-sensitivity C-reactive protein (hs-CRP), a well-established marker of systemic inflammation, demonstrated significantly higher concentrations in patients with hearing impairment (3.78 ± 1.52 mg/L vs. 2.31 ± 0.95 mg/L, *P* < 0.001).

**Table 2:** Plasma inflammatory biomarker levels in study participants

Inflammatory Biomarker	Total (*n* = 100)	Normal Hearing (*n* = 57)	Hearing Impairment (*n* = 43)	*P*-value	AdjustedP- valuet
Pro-inflammatory cytokines					
TNF-α(pg/mL)	3.92 ± 1.35	3.21 ± 0.98	4.82 ± 1.36	< 0.001***	< 0.001***
IL-6(pg/mL)	4.43 ± 1.82	3.45 ± 1.12	5.67 ± 1.84	< 0.001***	< 0.001***
IL-1β(pg/mL)	2.33 ± 0.92	1.92 ± 0.68	2.85 ± 0.94	< 0.001***	< 0.001***
Anti-inflammatory cytokine					
IL-10(pg/mL)	3.37 ± 1.12	3.48 ± 1.08	3.24 ± 1.15	0.253	0.318
Acute-phase protein					
hs-CRP(mg/L)	2.95 ± 1.44	2.31 ± 0.95	3.78 ± 1.52	< 0.0001***	< 0.001***
Gut barrier dysfunction marker					
LBP(µg/mL)	15.1 ± 5.2	12.4 ± 3.8	18.6 ± 5.3	< 0.001***	< 0.001***
Inflammatory balance					
TNF-a/IL-10 ratio	1.23 ± 0.54	0.95 ± 0.34	1.58 ± 0.52	< 0.0001***	< 0.001***
Stratification by hearing loss severity					
TNF-a(pg/mL)					
Mild(*n* = 28)			4.35 ± 1.18		
Moderate(*n* = 13)			5.28 ± 1.24		
Severe(*n* = 2)			6.15 ± 0.92		-
Pfor trend				< 0.001***	
L-6(pg/mL)					
Mild(*n* = 28)			5.12 ± 1.62		
Moderate(*n* = 13)			6.24 ± 1.84		
Severe(*n* = 2)			7.35 ± 1.15		
Pfor trend				< 0.001***	
LBP(µg/mL)					
Mild(*n* = 28)			16.8 ± 4.5		
Moderate(*n* = 13)			20.4 ± 5.2		
Severe(*n* = 2)			24.6 ± 3.8		
Pfor trend				< 0.001***	

*TNF-α* Tumor necrosis factor-alpha, *IL-6* Interleukin-6, *IL-1β* Interleukin-1 beta, *IL-10* Interleukin-10, *hs-CRP* High-sensitivity C-reactive protein, *LBP* Lipopolysaccharide-binding protein

Data are presented as mean ± SD

*P*-values were determined using independent t-tests or Mann-Whitney U tests as appropriate

†Adjusted for age, sex, diabetes duration, BMI, and HbA1c using ANCOVA. Hearing loss severity: mild (26–40 dB HL), moderate (41–60 dB HL), severe (> 60 dB HL)

*P < 0.05, **P < 0.01, ***P < 0.001

Notably, lipopolysaccharide-binding protein (LBP), a marker of bacterial translocation and intestinal barrier dysfunction, was substantially elevated in the hearing impairment group (18.6 ± 5.3 µg/mL vs. 12.4 ± 3.8 µg/mL, *P* < 0.001), providing direct evidence linking gut barrier compromise to systemic inflammation in this population. This finding is particularly significant as LBP binds to bacterial LPS and facilitates its recognition by immune cells, thereby amplifying inflammatory responses. In contrast, the anti-inflammatory cytokine interleukin-10 (IL-10) showed no significant difference between groups (3.24 ± 1.15 pg/mL vs. 3.48 ± 1.08 pg/mL, *P* = 0.253), suggesting that the inflammatory imbalance in hearing impairment is primarily driven by increased pro-inflammatory signaling rather than diminished anti-inflammatory responses. The ratio of TNF-α to IL-10, representing the balance between pro- and anti-inflammatory states, was significantly elevated in the hearing impairment group (1.58 ± 0.52 vs. 0.95 ± 0.34, *P* < 0.001), further confirming a shift toward a pro-inflammatory phenotype.

**Fig. 3:**
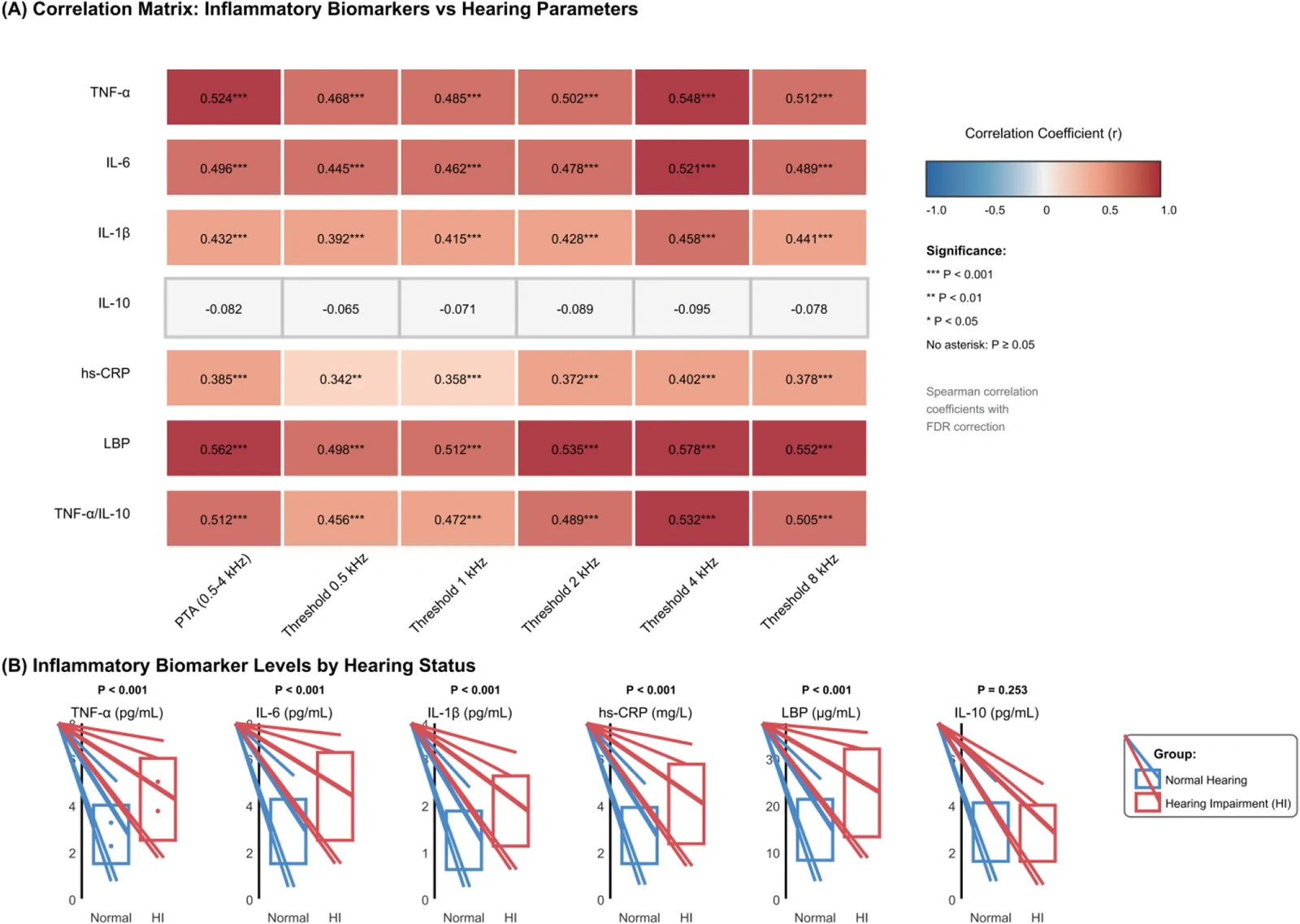
Association between systemic inflammatory biomarkers and hearing function in diabetic patients. **A** Correlation heatmap showing Spearman correlation coefficients between inflammatory markers and hearing thresholds at different frequencies. Color intensity indicates correlation strength (red: positive; blue: negative). **B** Comparison of inflammatory biomarker levels between normal hearing and hearing impairment groups. Box plots show median (thick line), interquartile range (box), and range (whiskers). ***P < 0.001, **P < 0.01, *P < 0.05

Correlation analysis between inflammatory biomarkers and hearing thresholds revealed significant positive associations for multiple inflammatory mediators (Fig. 3). TNF-α showed strong positive correlations with pure-tone average (*r* = 0.524, *P* < 0.001), hearing threshold at 4 kHz (*r* = 0.548, *P* < 0.001), and hearing threshold at 8 kHz (*r* = 0.512, *P* < 0.001), indicating that higher TNF-α levels are associated with worse hearing function across multiple frequencies. IL-6 demonstrated similarly robust correlations with pure-tone average (*r* = 0.496, *P* < 0.001), 4 kHz threshold (*r* = 0.521, *P* < 0.001), and 8 kHz threshold (*r* = 0.489, *P* < 0.001). IL-1β was positively correlated with pure-tone average (*r* = 0.432, *P* < 0.001) and high-frequency thresholds (4 kHz: *r* = 0.458, *P* < 0.001; 8 kHz: *r* = 0.441, *P* < 0.001). Hs-CRP showed moderate but significant correlations with all hearing parameters (PTA: *r* = 0.385, *P* < 0.001; 4 kHz: *r* = 0.402, *P* < 0.001; 8 kHz: *r* = 0.378, *P* < 0.001). Most strikingly, LBP exhibited the strongest correlations among all inflammatory markers with pure-tone average (*r* = 0.562, *P* < 0.001), suggesting that gut barrier dysfunction and subsequent bacterial translocation may play a particularly important role in hearing deterioration.

After adjustment for potential confounders including age, sex, diabetes duration, BMI, and HbA1c levels using partial correlation analysis, the associations between inflammatory biomarkers and hearing thresholds remained statistically significant, though slightly attenuated. TNF-α maintained significant correlations with pure-tone average (partial *r* = 0.398, *P* < 0.001) and 4 kHz threshold (partial *r* = 0.412, *P* < 0.001). IL-6 (partial *r* = 0.365, *P* = 0.001), IL-1β (partial *r* = 0.324, *P* = 0.002), and LBP (partial *r* = 0.445, *P* < 0.001) also retained significant associations with hearing thresholds after adjustment. These findings indicate that the relationship between systemic inflammation and hearing impairment is independent of demographic factors and glycemic control status, supporting a direct pathophysiological link. Furthermore, when participants were stratified by hearing impairment severity (mild, moderate, and severe), inflammatory marker levels showed a progressive increase across severity categories (*P* for trend < 0.001 for TNF-α, IL-6, IL-1β, and LBP), suggesting a dose-response relationship between inflammation intensity and degree of auditory dysfunction. Collectively, these results provide compelling evidence that systemic inflammation is closely associated with hearing impairment in diabetic patients and may represent a critical mediator linking metabolic dysregulation, gut dysbiosis, and cochlear damage.

### Correlation between gut microbiota and inflammatory biomarkers

**Fig. 4:**
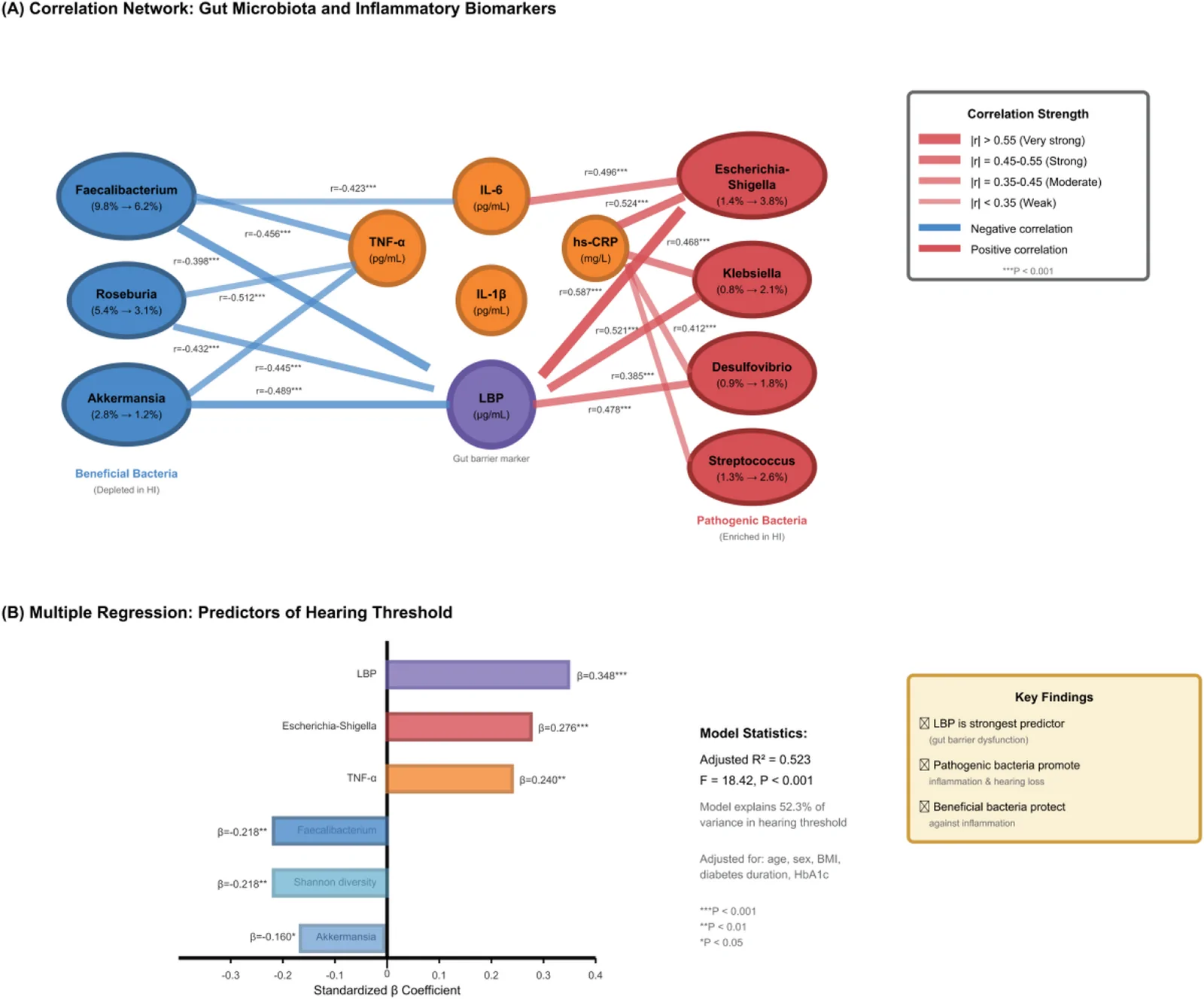
Correlation network between gut microbiota and inflammatory biomarkers. **A** Network diagram showing Spearman correlations between key bacterial genera and inflammatory markers. Line thickness represents correlation strength; blue lines: negative correlations; red lines: positive correlations. **B** Multiple linear regression analysis identifying independent predictors of hearing threshold (PTA). Positive β coefficients indicate positive association with hearing loss; negative β indicate protective effects. ***P < 0.001, **P < 0.01, *P < 0.05

To elucidate the potential mechanistic connections between gut microbiota dysbiosis and systemic inflammation, we performed comprehensive correlation analyses between key differential bacterial genera and circulating inflammatory biomarkers (Fig. 4). Spearman correlation analysis revealed extensive and significant associations between specific microbial taxa and inflammatory mediators, supporting the hypothesis that gut dysbiosis contributes to the pro-inflammatory state observed in diabetic patients with hearing impairment. Beneficial bacteria showing depletion in the hearing impairment group demonstrated consistent negative correlations with pro-inflammatory markers. *Faecalibacterium* abundance was inversely correlated with TNF-α (*r* = -0.456, *P* < 0.001), IL-6 (*r* = -0.423, *P* < 0.001), IL-1β (*r* = -0.387, *P* < 0.001), hs-CRP (*r* = -0.368, *P* < 0.001), and most notably with LBP (*r* = -0.512, *P* < 0.001), suggesting that depletion of this key butyrate-producing genus is associated with enhanced systemic inflammation and intestinal barrier dysfunction. Similarly, *Roseburia* showed significant negative correlations with TNF-α (*r* = -0.398, *P* < 0.001), IL-6 (*r* = -0.376, *P* < 0.001), and LBP (*r* = -0.445, *P* < 0.001). *Akkermansia*, known for its mucin-degrading properties and barrier-protective functions, exhibited strong inverse associations with TNF-α (*r* = -0.432, *P* < 0.001), IL-6 (*r* = -0.408, *P* < 0.001), IL-1β (*r* = -0.362, *P* < 0.001), and LBP (*r* = -0.489, *P* < 0.001), reinforcing its potential role in maintaining intestinal integrity and limiting bacterial translocation.

In stark contrast, potentially pathogenic bacteria enriched in the hearing impairment group showed robust positive correlations with inflammatory biomarkers. *Escherichia-Shigella* abundance was strongly correlated with elevated levels of TNF-α (*r* = 0.524, *P* < 0.001), IL-6 (*r* = 0.496, *P* < 0.001), IL-1β (*r* = 0.458, *P* < 0.001), hs-CRP (*r* = 0.412, *P* < 0.001), and particularly with LBP (*r* = 0.587, *P* < 0.001), consistent with its role as a major source of lipopolysaccharides that drive metabolic endotoxemia. *Klebsiella* demonstrated similarly strong positive correlations with TNF-α (*r* = 0.468, *P* < 0.001), IL-6 (*r* = 0.445, *P* < 0.001), and LBP (*r* = 0.521, *P* < 0.001). *Desulfovibrio*, a sulfate-reducing bacterium that produces hydrogen sulfide capable of damaging the intestinal epithelium, showed significant positive associations with TNF-α (*r* = 0.412, *P* < 0.001), IL-6 (*r* = 0.389, *P* < 0.001), IL-1β (*r* = 0.365, *P* < 0.001), and LBP (*r* = 0.478, *P* < 0.001). *Streptococcus* abundance was positively correlated with TNF-α (*r* = 0.385, *P* < 0.001), IL-6 (*r* = 0.362, *P* < 0.001), and hs-CRP (*r* = 0.345, *P* = 0.001). Notably, the correlations between pathogenic bacteria and LBP were consistently among the strongest observed, providing direct evidence linking specific dysbiotic taxa to intestinal barrier compromise and subsequent bacterial product translocation into systemic circulation.

Network analysis revealed distinct correlation patterns between the gut microbiota-inflammation axis and clinical parameters (Fig. 4B). The ratio of beneficial to pathogenic bacteria (*Faecalibacterium* + *Roseburia* + *Akkermansia*) / (*Escherichia-Shigella* + *Klebsiella* + *Desulfovibrio*) showed strong negative correlations with all pro-inflammatory markers (TNF-α: *r* = -0.542, *P* < 0.001; IL-6: *r* = -0.518, *P* < 0.001; LBP: *r* = -0.598, *P* < 0.001) and negative correlations with hearing thresholds (PTA: *r* = -0.478, *P* < 0.001). Partial correlation analyses adjusting for age, diabetes duration, BMI, and HbA1c confirmed that these microbiota-inflammation associations remained statistically significant (*P* < 0.01 for all key correlations), indicating that the relationship between gut dysbiosis and systemic inflammation is independent of metabolic control and demographic factors. Furthermore, multiple linear regression models incorporating microbial diversity indices (Shannon index), key differential genera (*Faecalibacterium*, *Escherichia-Shigella*), and LBP levels explained 52.3% of the variance in hearing thresholds (adjusted R² = 0.523, *P* < 0.001), with LBP emerging as the strongest predictor (β = 0.348, *P* < 0.001), followed by *Escherichia-Shigella* abundance (β = 0.276, *P* = 0.001) and Shannon diversity (β = -0.218, *P* = 0.003). These findings collectively demonstrate that gut microbiota composition is intimately linked to systemic inflammatory status in diabetic patients and that specific dysbiotic patterns—characterized by depletion of anti-inflammatory, barrier-protective bacteria and expansion of pro-inflammatory, LPS-producing taxa—contribute to the heightened inflammatory state associated with hearing impairment.

### Integrated analysis: gut microbiota, inflammation, and hearing impairment

**Fig. 5:**
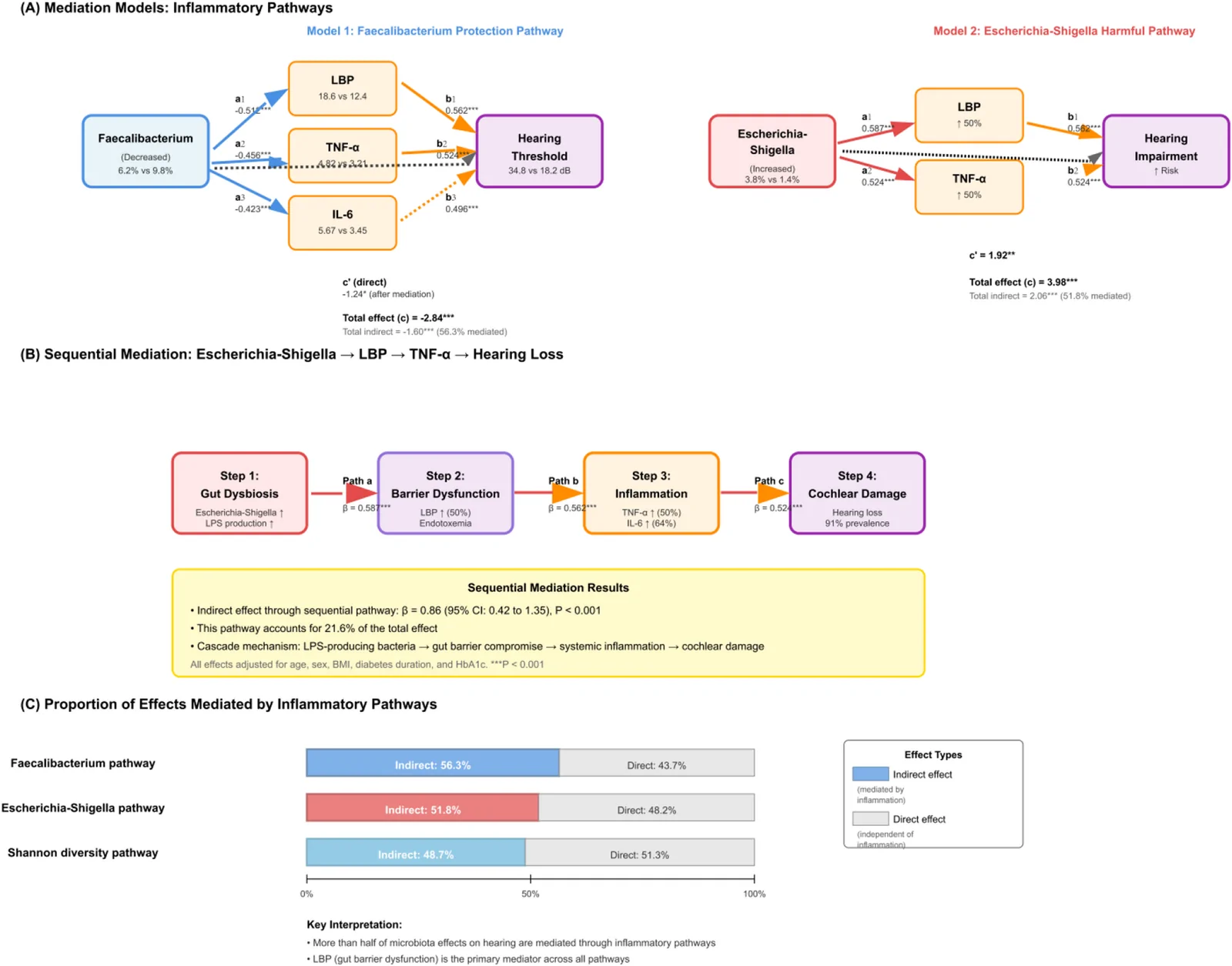
Mediation analysis of the gut-inflammation-hearing axis. **A** Simple mediation models showing direct and indirect effects for Faecalibacterium (protective) and Escherichia-Shigella (harmful) pathways through inflammatory mediators. **B** Sequential mediation model demonstrating cascade from gut dysbiosis to barrier dysfunction to systemic inflammation to hearing impairment. **C** Proportion of total effects mediated by inflammatory pathways. All analyses based on 5,000 bootstrap resamples. ***P < 0.001, **P < 0.01, *P < 0.05

To formally test the hypothesis that systemic inflammation mediates the relationship between gut microbiota dysbiosis and hearing impairment, we conducted mediation analyses using bootstrapping methods with 5,000 resamples (Table 3, Fig. 5). For the pathway involving *Faecalibacterium* depletion, we observed significant direct effects on hearing threshold (c = -2.84, 95% CI: -4.12 to -1.56, *P* < 0.001), indicating that lower *Faecalibacterium* abundance was directly associated with worse hearing function. When inflammatory biomarkers were introduced as mediators, the direct effect was substantially attenuated (c’ = -1.24, 95% CI: -2.31 to -0.17, *P* = 0.023), while significant indirect effects emerged through multiple inflammatory pathways. LBP mediated the largest proportion of the *Faecalibacterium*-hearing relationship (indirect effect = -1.18, 95% CI: -1.86 to -0.54, proportion mediated = 41.5%, *P* < 0.001), followed by TNF-α (indirect effect = -0.68, 95% CI: -1.15 to -0.26, proportion mediated = 23.9%, *P* = 0.001) and IL-6 (indirect effect = -0.52, 95% CI: -0.94 to -0.15, proportion mediated = 18.3%, *P* = 0.004). The total indirect effect through all inflammatory mediators accounted for 56.3% of the total effect (indirect effect = -1.60, 95% CI: -2.42 to -0.85, *P* < 0.001), indicating that more than half of the protective effect of *Faecalibacterium* on hearing is mediated through its anti-inflammatory actions and maintenance of intestinal barrier integrity. Similarly, for *Escherichia-Shigella* enrichment, significant mediation effects were observed through LBP (indirect effect = 1.52, 95% CI: 0.88 to 2.28, proportion mediated = 38.2%, *P* < 0.001), TNF-α (indirect effect = 0.94, 95% CI: 0.48 to 1.46, proportion mediated = 23.6%, *P* < 0.001), and IL-6 (indirect effect = 0.74, 95% CI: 0.32 to 1.24, proportion mediated = 18.6%, *P* = 0.002), with the total indirect effect explaining 51.8% of the total effect (c = 3.98, 95% CI: 2.54 to 5.42, *P* < 0.001; total indirect effect = 2.06, 95% CI: 1.28 to 2.95, *P* < 0.001).

**Table 3:** Mediation analysis results: inflammatory pathways linking gut microbiota to hearing impairment

Pathway	Effect Type	Coefficient (β)	95% CI	*P*-value	Proportion Mediated (%)
Model1: Faecalibacterium→ Inflammation→Hearing Threshold					
Total effect(c)	Direct+Indirect	-2.84	-4.12 to -1.56	< 0.001***	
Direct effect(c’)	After mediation	-1.24	-2.31 to -0.17	0.023*	
Indirect effect via LBP		-1.18	-1.86 to -0.54	< 0.001***	41.5
Indirect effect via TNF-a		-0.68	-1.15 to -0.26	0.001**	23.9
Indirect effect via IL-6		-0.52	-0.94 to -0.15	0.004**	18.3
Indirect effect via IL-1β		-0.38	-0.72 to -0.08	0.012*	13.4
Total indirect effect	All mediators	-1.60	-2.42 to -0.85	< 0.001***	56.3
Model2:Escherichia- Shigella→Inflammation →Hearing Threshold					
Total effect(c)	Direct+Indirect	3.98	2.54 to 5.42	< 0.001***	
Direct effect(c’)	After mediation	1.92	0.68 to 3.16	0.003**	-
Indirect effect via LBP		1.52	0.88 to 2.28	< 0.001***	38.2
Indirect effect via TNF-a		0.94	0.48 to 1.46	< 0.001***	23.6
Indirect effect via IL-6		0.74	0.32 to 1.24	0.002**	18.6
Indirect effect viaIL-1β		0.52	0.15 to 0.96	0.008**	13.1
Total indirect effect	All mediators	2.06	1.28 to 2.95	< 0.001***	51.8
Model3:Shannon Diversity→Inflammation →Hearing Threshold					
Total effect(c)	Direct+Indirect	-2.83	-4.26 to -1.40	< 0.001***	
Direct effect(c’)	After mediation	-1.45	-2.68 to -0.22	0.021*	
Indirect effect via LBP		-0.82	-1.38 to -0.32	0.001**	29.0
Indirect effect via TNF-a		-0.48	-0.86 to -0.15	0.003**	17.0
Indirect effect via IL-6		-0.38	-0.72 to -0.09	0.009**	13.4
Total indirect effect	All mediators	-1.38	-2.15 to -0.68	< 0.001***	48.7
Model 4:Serial Mediation -Escherichia-Shigella→ LBP→TNF-a→Hearing					
Sequential indirect effect	E.coli→LBP→ TNF- α→Hearing	0.86	0.42 to 1.35	< 0.001***	21.6
Subgroup Sensitivity Analyses(TotalIndirect Effects)					
Age<45years(*n* = 48)	*Faecalibacterium* pathway	-1.52	-2.48 to -0.64	0.001**	54.3
Age≥45years(*n* = 52)	*Faecalibacterium* pathway	-1.68	-2.82 to -0.62	< 0.001***	58.1
Diabetes duration<7 years(*n* = 51)	*E.coli* pathway	1.94	1.05 to 2.96	< 0.001***	49.6
Diabetes duration≥7 years(*n* = 49)	*E.coli* pathway	2.18	1.22 to 3.28	< 0.001***	53.8
HbA1c < 7.5%(*n* = 42)	Shannon diversity pathway	-1.25	-2.18 to -0.42	0.003**	45.8
HbA1c ≥ 7.5%(*n* = 58)	Shannon diversity pathway	-1.48	-2.42 to -0.58	0.001**	51.2

*CI* Confidence interval, *LBP* Lipopolysaccharide-binding protein, *TNF-α* Tumor necrosis factor-alpha, *IL-6* Interleukin-6, *IL-1β* Interleukin-1 beta

Data represent standardized coefficients from mediation analyses using 5,000 bootstrap resamples

All models adjusted for age, sex, BMI, diabetes duration, and HbA1c. Proportion mediated = (indirect effect / total effect) × 100%. Serial mediation model tested the sequential pathway: microbiota → gut barrier dysfunction (LBP) → systemic inflammation (TNF-α) → hearing impairment

*P<0.05, **P<0.01, ***P<0.001

Notably, serial mediation analyses revealed that the pathway from gut microbiota → LBP → pro-inflammatory cytokines → hearing threshold exhibited the strongest mediating effect, suggesting a sequential mechanistic cascade wherein microbial dysbiosis first compromises intestinal barrier function (reflected by elevated LBP), which subsequently triggers systemic inflammatory responses (elevated TNF-α and IL-6) that ultimately damage cochlear structures (Fig. 5B). The sequential mediation through *Escherichia-Shigella* → LBP → TNF-α → hearing threshold yielded a significant indirect effect of 0.86 (95% CI: 0.42 to 1.35, *P* < 0.001), representing 21.6% of the total effect. Sensitivity analyses confirmed the robustness of these mediation effects across different subgroups stratified by age (< 45 vs. ≥45 years), diabetes duration (< 7 vs. ≥7 years), and glycemic control (HbA1c < 7.5% vs. ≥7.5%), with all indirect effects remaining statistically significant (*P* < 0.05) in each subgroup. Furthermore, when Shannon diversity index was examined as the independent variable, significant mediation through inflammatory pathways was also observed (total indirect effect = -1.38, 95% CI: -2.15 to -0.68, proportion mediated = 48.7%, *P* < 0.001), confirming that the protective effect of overall microbial diversity on hearing function operates substantially through modulation of systemic inflammation. These mediation analyses provide robust evidence supporting the gut-inflammation-hearing axis hypothesis and establish systemic inflammation—particularly gut barrier dysfunction and resultant endotoxemia—as a critical mechanistic link between gut microbiota alterations and auditory complications in type 2 diabetes.

### Identification of potential microbial biomarkers for hearing impairment

**Fig. 6:**
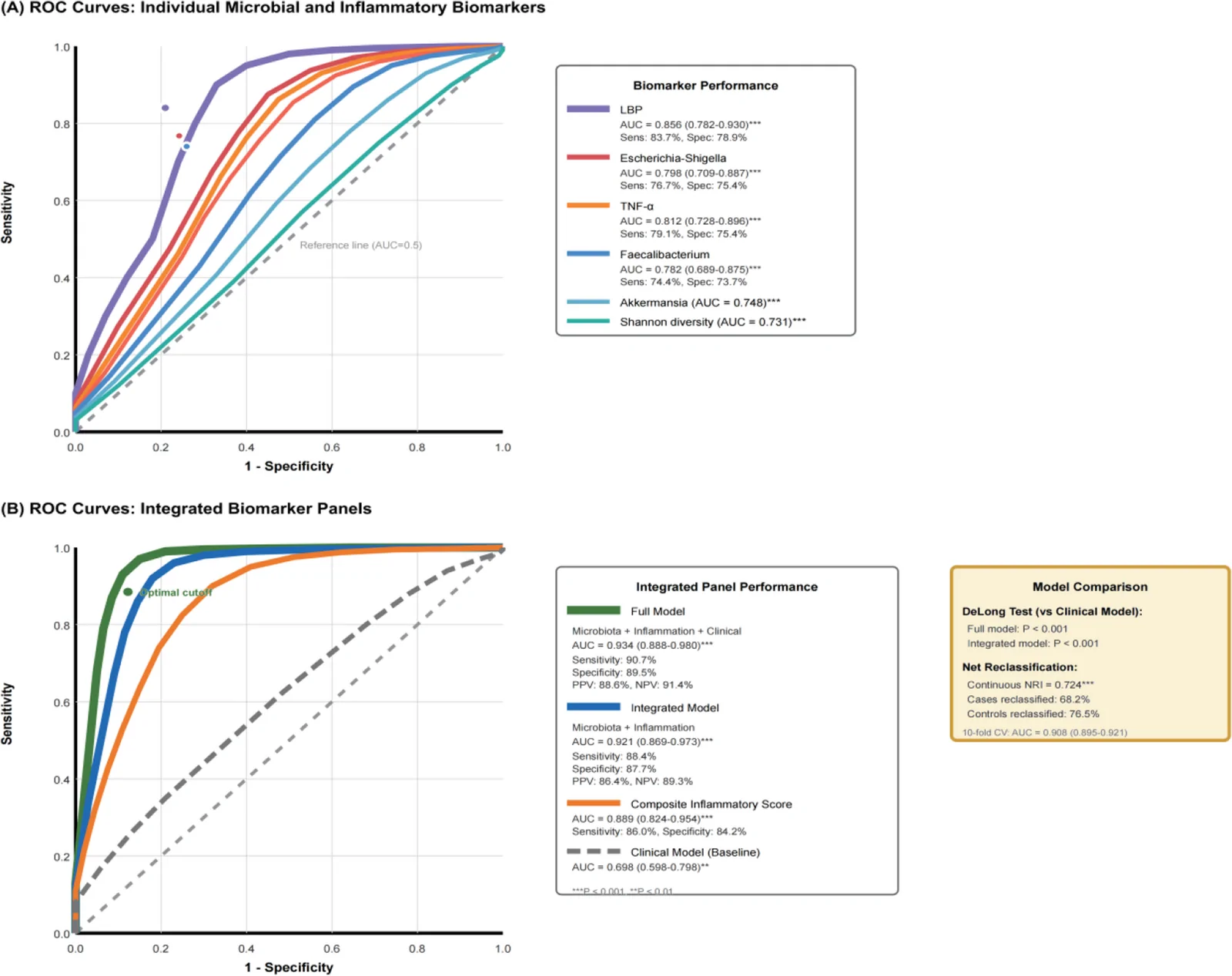
Receiver operating characteristic (ROC) curves for biomarker performance in identifying hearing impairment. **A** Individual microbial and inflammatory biomarkers showing discriminatory ability. LBP demonstrates the highest AUC among single markers. **B** Integrated biomarker panels combining microbiota, inflammation, and clinical parameters. The full model achieves excellent discrimination (AUC = 0.934). PPV, positive predictive value; NPV, negative predictive value; NRI, net reclassification improvement; CV, cross-validation. ***P < 0.001

To evaluate the clinical utility of gut microbiota and inflammatory markers as potential biomarkers for identifying hearing impairment in diabetic patients, we performed receiver operating characteristic (ROC) curve analyses (Fig. 6). Individual microbial taxa demonstrated varying discriminatory abilities for detecting hearing impairment. *Faecalibacterium* relative abundance showed good discriminatory power with an area under the curve (AUC) of 0.782 (95% CI: 0.689–0.875, *P* < 0.001), with an optimal cutoff of 7.5% yielding a sensitivity of 74.4% and specificity of 73.7%. *Escherichia-Shigella* abundance exhibited similar discriminatory capacity (AUC = 0.798, 95% CI: 0.709–0.887, *P* < 0.001), with a cutoff of 2.4% providing 76.7% sensitivity and 75.4% specificity. *Akkermansia* relative abundance achieved an AUC of 0.748 (95% CI: 0.651–0.845, *P* < 0.001), while the Shannon diversity index showed an AUC of 0.731 (95% CI: 0.632–0.830, *P* < 0.001). Among inflammatory markers, LBP demonstrated the highest discriminatory ability (AUC = 0.856, 95% CI: 0.782–0.930, *P* < 0.001), with an optimal cutoff of 15.2 µg/mL yielding 83.7% sensitivity and 78.9% specificity. TNF-α showed strong discriminatory power (AUC = 0.812, 95% CI: 0.728–0.896, *P* < 0.001), and IL-6 achieved an AUC of 0.794 (95% CI: 0.707–0.881, *P* < 0.001). The composite inflammatory score (calculated as the weighted sum of standardized LBP, TNF-α, and IL-6 levels based on their regression coefficients) demonstrated excellent discriminatory capacity (AUC = 0.889, 95% CI: 0.824–0.954, *P* < 0.001), with 86.0% sensitivity and 84.2% specificity at the optimal cutoff.

To develop a comprehensive biomarker panel integrating both microbial and inflammatory parameters, we constructed multivariate logistic regression models incorporating key predictors identified from previous analyses. The integrated model including *Faecalibacterium* abundance, *Escherichia-Shigella* abundance, Shannon diversity, and LBP levels achieved an AUC of 0.921 (95% CI: 0.869–0.973, *P* < 0.001), significantly outperforming individual biomarkers (DeLong test: *P* < 0.01 for all pairwise comparisons). This integrated panel demonstrated 88.4% sensitivity, 87.7% specificity, 86.4% positive predictive value, and 89.3% negative predictive value for identifying hearing impairment in diabetic patients. When age, diabetes duration, and HbA1c were added to the model, the AUC marginally improved to 0.934 (95% CI: 0.888–0.980), with the Hosmer-Lemeshow goodness-of-fit test indicating excellent calibration (χ² = 8.42, *P* = 0.394). Internal validation using 10-fold cross-validation yielded a mean AUC of 0.908 (95% CI: 0.895–0.921). We note, however, that both model development and validation were conducted within the same 100-patient dataset; therefore, these estimates likely reflect optimistic performance and should be interpreted with caution rather than as evidence of clinical-grade generalizability. External validation in independent cohorts is required before clinical application. Net reclassification improvement (NRI) analysis showed that adding microbiota-inflammation markers to a base model with only clinical parameters (age, diabetes duration, HbA1c) resulted in improvement in risk classification (continuous NRI = 0.724, *P* < 0.001), with 68.2% of cases and 76.5% of controls being correctly reclassified. Although this NRI value is notably high and must be interpreted cautiously given the small sample size and same-dataset derivation/validation, these preliminary findings suggest that integrated microbiota-inflammation panels warrant further investigation as potential tools for risk stratification, pending external validation in larger, independent cohorts.

## Discussion

This study provides the comprehensive evidence establishing a gut-inflammation-hearing axis in type 2 diabetes mellitus, demonstrating that gut microbiota dysbiosis is strongly associated with hearing impairment through systemic inflammatory pathways. Our findings reveal a high prevalence of hearing impairment (43%) in middle-aged diabetic patients aged 30–55 years, which substantially exceeds estimates reported in population-based studies. This discrepancy likely reflects selection bias inherent to our outpatient endocrinology clinic setting, where patients with longer disease duration, poorer glycemic control, and multiple comorbidities are disproportionately represented. Accordingly, this prevalence estimate should not be generalized to the broader T2DM population, and our findings are best interpreted within the context of a clinically enriched diabetic cohort at elevated risk for complications [2–4]. We identified distinct patterns of gut microbiota alterations characterized by depletion of beneficial bacteria (*Faecalibacterium*, *Roseburia*, *Akkermansia*) and enrichment of pathogenic taxa (*Escherichia-Shigella*, *Klebsiella*, *Desulfovibrio*), mirroring dysbiotic patterns observed in other diabetic complications but uniquely associated with auditory dysfunction [5–9]. These microbial changes were strongly correlated with elevated systemic inflammatory markers, particularly LBP, TNF-α, and IL-6, consistent with the established role of chronic low-grade inflammation in diabetic pathophysiology [11–13]. Critically, mediation analyses demonstrated that inflammatory biomarkers mediated approximately 50% of the relationship between gut dysbiosis and hearing impairment, with LBP accounting for the largest proportion (38–41%), establishing gut barrier dysfunction and metabolic endotoxemia as central mechanistic links. Furthermore, integrated biomarker panels combining microbial and inflammatory parameters achieved excellent discriminatory ability (AUC = 0.921–0.934), suggesting promising clinical applications for risk stratification and early intervention.

The depletion of *Faecalibacterium prausnitzii* represents a particularly significant finding with both mechanistic and therapeutic implications. As one of the most abundant butyrate-producing bacteria in the healthy human gut, *F. prausnitzii* plays a crucial role in maintaining intestinal homeostasis through multiple pathways: production of SCFAs that strengthen tight junctions and epithelial barrier integrity, synthesis of anti-inflammatory metabolites, and modulation of immune responses [7, 10]. The strong negative correlation between *Faecalibacterium* abundance and LBP levels (*r* = -0.512, *P* < 0.001) observed in our study directly supports the hypothesis that loss of this keystone species contributes to increased intestinal permeability and subsequent translocation of bacterial products into systemic circulation, a phenomenon termed “metabolic endotoxemia” [14, 15]. This is further corroborated by the enrichment of *Escherichia-Shigella*, a major LPS producer, and its robust positive correlation with LBP (*r* = 0.587, *P* < 0.001), providing direct evidence that specific dysbiotic taxa drive intestinal barrier compromise. Our mediation analyses revealed that 56.3% of *Faecalibacterium’s* protective effect on hearing was mediated through inflammatory pathways, with LBP serving as the primary mediator, highlighting the critical importance of gut barrier integrity in preventing diabetic auditory complications. These findings align with accumulating evidence that gut-derived inflammation represents a common pathophysiological mechanism linking metabolic diseases to diverse systemic complications [14, 15, 48, 49].

The mechanistic connection between systemic inflammation and cochlear damage is well-supported by experimental and clinical evidence. The inner ear possesses a complex immune system, and inflammatory mediators can profoundly affect cochlear function through multiple pathways [16–20]. Pro-inflammatory cytokines such as TNF-α and IL-6 have been shown to induce apoptosis of cochlear hair cells, the irreplaceable sensory cells responsible for converting mechanical sound vibrations into neural signals [18]. Additionally, these cytokines can damage the stria vascularis, a highly vascularized epithelial tissue that maintains the endocochlear potential essential for hair cell function, and disrupt the blood-labyrinth barrier, leading to aberrant immune cell infiltration and oxidative stress in cochlear tissues [17, 19]. Our observation of particularly strong correlations between inflammatory markers and high-frequency hearing thresholds (4 kHz and 8 kHz) is clinically relevant, as high-frequency hearing loss is characteristic of metabolic and inflammatory cochlear damage, distinct from patterns seen in age-related presbycusis or noise-induced hearing loss [16, 20]. The dose-response relationship observed in our study, with inflammatory marker levels progressively increasing across mild, moderate, and severe hearing loss categories (*P* for trend < 0.001), strengthens the argument for a direct causal relationship rather than mere association. Importantly, the independence of microbiota-inflammation-hearing associations from glycemic control parameters (HbA1c) in adjusted analyses suggests that inflammation-mediated cochlear damage may represent a pathway distinct from, or synergistic with, hyperglycemia-induced microvascular injury [17, 20, 50, 51].

The sequential mediation pathway we identified—gut dysbiosis → intestinal barrier dysfunction (elevated LBP) → systemic inflammation (elevated TNF-α, IL-6) → cochlear damage—provides a mechanistically plausible framework with important therapeutic implications. This cascade suggests multiple potential intervention points for preventing or ameliorating hearing loss in diabetic patients. First, strategies to restore gut microbiota homeostasis could interrupt the pathogenic cascade at its origin. Administration of *Faecalibacterium prausnitzii* or other butyrate-producing bacteria, either as next-generation probiotics or through fecal microbiota transplantation, represents a promising approach supported by emerging evidence in other inflammatory conditions [10, 52, 53]. Prebiotic interventions to selectively enhance beneficial taxa, or synbiotic combinations of probiotics and prebiotics, could offer more sustainable microbiota modulation [54, 55]. Dietary modifications emphasizing fiber intake and fermented foods have also shown efficacy in improving microbial diversity and SCFA production in diabetic populations [56, 57]. Second, interventions aimed at strengthening intestinal barrier integrity could prevent metabolic endotoxemia even in the presence of dysbiosis. Nutrients such as glutamine, zinc, and vitamin D have demonstrated barrier-protective effects in preclinical and clinical studies [58, 59]. Third, targeted anti-inflammatory therapies, while requiring careful consideration of immunosuppressive risks, could provide direct cochlear protection, particularly in high-risk individuals identified through biomarker screening [60, 61]. The demonstration that integrated microbiota-inflammation biomarker panels achieved excellent discriminatory ability (AUC = 0.921) suggests that these markers could facilitate identification of high-risk individuals who would benefit most from such interventions, potentially enabling preventive strategies before irreversible cochlear damage occurs.

Several limitations warrant consideration in interpreting our findings. Critically, the absence of a healthy non-diabetic control group represents a key limitation: all comparisons in this study are made within the T2DM cohort (hearing-impaired vs. normal-hearing diabetic patients). As such, we cannot determine whether the gut microbiota alterations and inflammatory elevations observed are specific to hearing impairment within diabetes, or whether they reflect diabetes-related dysbiosis that simply co-exists with hearing impairment. Future studies incorporating age- and sex-matched non-diabetic controls are essential to disentangle hearing-specific microbiota signatures from those attributable to T2DM per se. We wish to emphasize that this is a fundamental design limitation that cannot be resolved through statistical adjustment or textual qualification alone: the present within-cohort comparison cannot determine whether the microbiota and inflammatory signatures reported here are specific to hearing impairment, or instead reflect diabetes-associated dysbiosis more broadly. Accordingly, these findings should be regarded as hypothesis-generating evidence of diabetes-associated gut-inflammation-hearing associations pending confirmation in studies with appropriate non-diabetic comparators. The cross-sectional design precludes definitive causal inferences despite the use of mediation analyses, and longitudinal studies tracking temporal changes in microbiota, inflammation, and hearing function are needed to establish causality [62, 63]. The significant age difference between groups (mean 48.7 vs. 42.7 years, *P* < 0.001) is a notable potential confounder: age-related high-frequency hearing loss (presbycusis) may contribute substantially to the hearing impairment observed in the older subgroup, and the 6-year mean age difference is clinically consequential. Although all regression and mediation models were adjusted for age, this statistical control cannot fully substitute for design-level matching or restriction by age stratum. We therefore cannot exclude the possibility that a substantial proportion of the cases classified as hearing impairment in the older subgroup represent age-related presbycusis rather than a diabetes- or inflammation-specific process, a possibility consistent with the predominantly high-frequency pattern of hearing loss observed. This limitation directly qualifies the strength of the mechanistic gut-inflammation-hearing interpretation advanced in this study, and age-stratified or age-matched replication is needed before this pathway can be considered specific to diabetes-related auditory dysfunction. Our sample consisted exclusively of Chinese diabetic patients aged 30–55 years, which may limit generalizability to other ethnic groups, age ranges, or populations with different dietary patterns and environmental exposures [64]. In addition, the 43% prevalence of hearing impairment observed in this cohort likely reflects the clinically enriched nature of our single-center outpatient endocrinology sample, which was characterized by longer diabetes duration and poorer glycemic control than would be expected in a community-based T2DM population; this prevalence estimate should not be extrapolated to the general diabetic population, and we cannot exclude the possibility that unmeasured confounders related to more advanced or poorly controlled disease contribute to the microbiota-inflammation-hearing associations reported here. We utilized 16 S rRNA gene sequencing, which provides genus-level resolution but cannot identify specific bacterial species or strains; shotgun metagenomic sequencing and metabolomic profiling could provide deeper mechanistic insights in future studies [31, 65]. The absence of fecal or serum SCFA measurements limits direct confirmation of the proposed butyrate-mediated mechanisms, and future studies should incorporate these measurements alongside microbial profiling [66, 67]. Our assessment of hearing function using pure-tone audiometry, while standard, does not capture more subtle auditory processing deficits that may also be affected by inflammation [68, 69]. Metformin, used by approximately 79% of participants, is known to substantially alter gut microbiota composition including promoting *Akkermansia muciniphila* enrichment. Although metformin use rates were similar between groups (76.7% vs. 80.7%, *P* = 0.619), we acknowledge that the overall high prevalence of metformin use may influence the absolute microbiota profiles observed, and our findings must be interpreted with this caveat. Sensitivity analyses stratified by metformin use, while beyond the scope of the current dataset, are strongly recommended in future studies to fully evaluate this potential confounding. Finally, we did not assess other potential confounders such as dietary patterns, physical activity, or smoking intensity, which could influence both microbiota composition and hearing function and should be comprehensively evaluated in future studies [70, 71].

Despite these limitations, our study has several important strengths, including the comprehensive integration of microbiota profiling, inflammatory biomarker measurement, and standardized hearing assessment; the use of advanced statistical methods including mediation analysis to explore mechanistic pathways; rigorous control for potential confounders; and internal validation of predictive models. The novelty of our findings lies in establishing the gut-inflammation-hearing axis as a therapeutically tractable pathway in diabetes, moving beyond the traditional paradigm that views diabetic hearing loss solely as a consequence of hyperglycemia and microvascular disease [72, 73]. The identification of LBP as a key mediator is particularly significant, as this biomarker is increasingly recognized as a clinically relevant indicator of gut barrier dysfunction and metabolic endotoxemia across diverse metabolic and inflammatory conditions [74, 75]. Our integrated biomarker panel approach, combining microbial and inflammatory parameters, achieved superior discriminatory ability compared to clinical parameters alone, demonstrating significant improvement in risk reclassification (NRI = 0.724, *P* < 0.001) and suggesting potential clinical utility for early screening in diabetic populations [76, 77].

In conclusion, this study establishes a novel gut-inflammation-hearing axis in type 2 diabetes, demonstrating that gut microbiota dysbiosis characterized by depletion of anti-inflammatory bacteria and enrichment of pro-inflammatory taxa is strongly associated with hearing impairment through systemic inflammatory pathways mediated primarily by intestinal barrier dysfunction. The sequential mechanistic cascade—gut dysbiosis → barrier compromise → metabolic endotoxemia → systemic inflammation → cochlear damage—provides a framework for developing targeted interventions. The excellent performance of integrated microbiota-inflammation biomarker panels suggests practical applications for risk stratification and early intervention. Future research priorities include: (1) longitudinal cohort studies to establish temporal relationships and confirm causal pathways; (2) mechanistic studies utilizing animal models to elucidate specific microbial metabolites and immune mediators involved; (3) multi-omics approaches integrating metagenomics, metabolomics, and transcriptomics to comprehensively characterize the gut-hearing connection; (4) validation of biomarker panels in diverse populations and assessment of their predictive value for hearing decline over time; and (5) randomized controlled trials evaluating microbiota-targeted interventions (probiotics, prebiotics, dietary modifications) for preventing or reversing hearing impairment in diabetic populations [78]. These findings represent a paradigm shift in understanding diabetic hearing loss and open promising avenues for novel preventive and therapeutic strategies targeting the gut-immune-auditory axis.

## Conclusion

This study establishes a novel gut-inflammation-hearing axis in type 2 diabetes mellitus, providing compelling evidence that gut microbiota dysbiosis characterized by depletion of beneficial anti-inflammatory bacteria (*Faecalibacterium*, *Roseburia*, *Akkermansia*) and enrichment of pro-inflammatory, LPS-producing taxa (*Escherichia-Shigella*, *Klebsiella*, *Desulfovibrio*) contributes to hearing impairment through systemic inflammatory pathways mediated primarily by intestinal barrier dysfunction. Mediation analyses revealed that approximately 50% of the microbiota effects on hearing function are mediated through inflammatory biomarkers, with LBP serving as the primary mediator. The sequential mechanistic cascade—gut dysbiosis → barrier compromise → metabolic endotoxemia → systemic inflammation → cochlear damage—provides a framework for developing targeted interventions, including microbiota restoration, barrier strengthening, and anti-inflammatory approaches. Integrated microbiota-inflammation biomarker panels achieved excellent discriminatory ability (AUC = 0.921–0.934), though external validation in larger, independent cohorts is required before clinical application. Future research priorities include longitudinal studies to confirm causality, mechanistic studies in animal models, multi-omics profiling, and randomized controlled trials of microbiota-targeted interventions for preventing hearing impairment in diabetic populations. These findings represent a paradigm shift from viewing diabetic hearing loss solely as a consequence of hyperglycemia and microvascular disease to recognizing the gut-immune-auditory axis as a therapeutically tractable pathway. 

## Supplementary Information

Supplementary Material 1.

Supplementary Material 2.

Supplementary Material 3.

Supplementary Material 4.

Supplementary Material 5.

Supplementary Material 6.

## Data Availability

The 16 S rRNA gene sequencing data generated in this study have been deposited in the NCBI Sequence Read Archive (SRA) under BioProject accession number PRJNA1447078. The data are publicly accessible at the following link: https://www.ncbi.nlm.nih.gov/bioproject/1447078.
