# Supplementary material for: The gut-inflammation-hearing axis in diabetic patients aged 30–55: exploring microbiome-mediated pathways to auditory complications
Source: BMC Microbiol. 2026 Aug 15;26:995. doi: 10.1186/s12866-026-05504-8 (PMC13753257; doi:10.1186/s12866-026-05504-8)
Supplement: Supplementary file 1 [file 12866_2026_5504_MOESM1_ESM.pdf]

(A) Mediation Models: Inflammatory Pathways

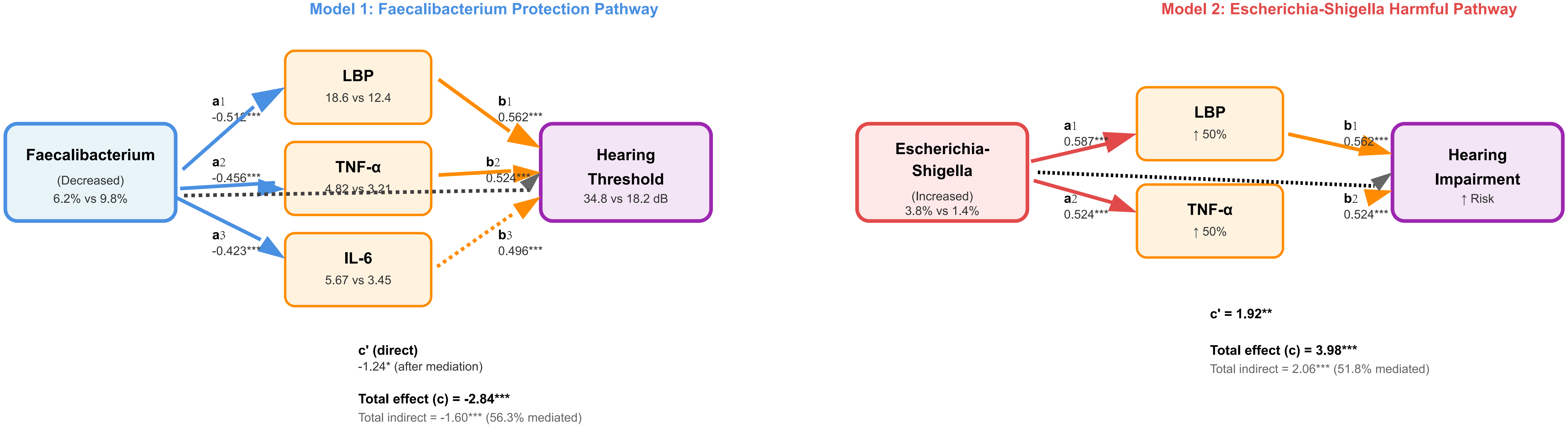

(B) Sequential Mediation: Escherichia-Shigella → LBP → TNF-α → Hearing Loss

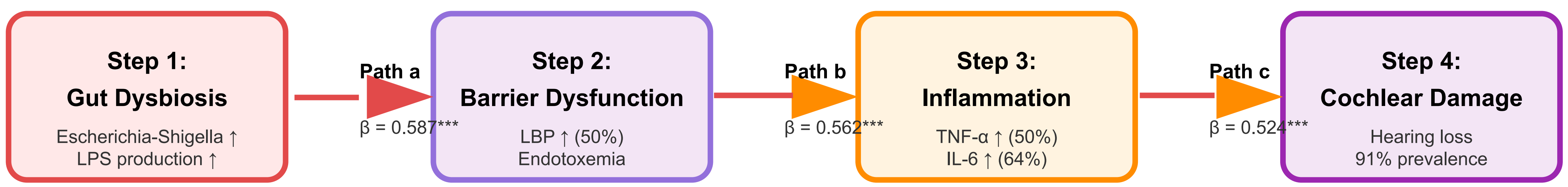

Sequential Mediation Results

- Indirect effect through sequential pathway: β = 0.86 (95% CI: 0.42 to 1.35), P < 0.001
- This pathway accounts for 21.6% of the total effect
- Cascade mechanism: LPS-producing bacteria → gut barrier compromise → systemic inflammation → cochlear damage

All effects adjusted for age, sex, BMI, diabetes duration, and HbA1c. ***P < 0.001

(C) Proportion of Effects Mediated by Inflammatory Pathways

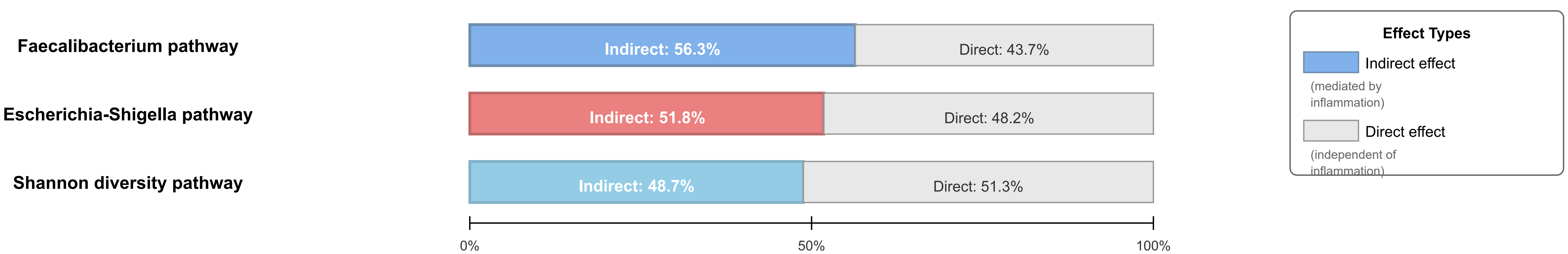

Key Interpretation:

- More than half of microbiota effects on hearing are mediated through inflammatory pathways
- LBP (gut barrier dysfunction) is the primary mediator across all pathways
