# Supplementary material for: The gut-inflammation-hearing axis in diabetic patients aged 30–55: exploring microbiome-mediated pathways to auditory complications
Source: BMC Microbiol. 2026 Aug 15;26:995. doi: 10.1186/s12866-026-05504-8 (PMC13753257; doi:10.1186/s12866-026-05504-8)
Supplement: Supplementary file 2 [file 12866_2026_5504_MOESM2_ESM.pdf]

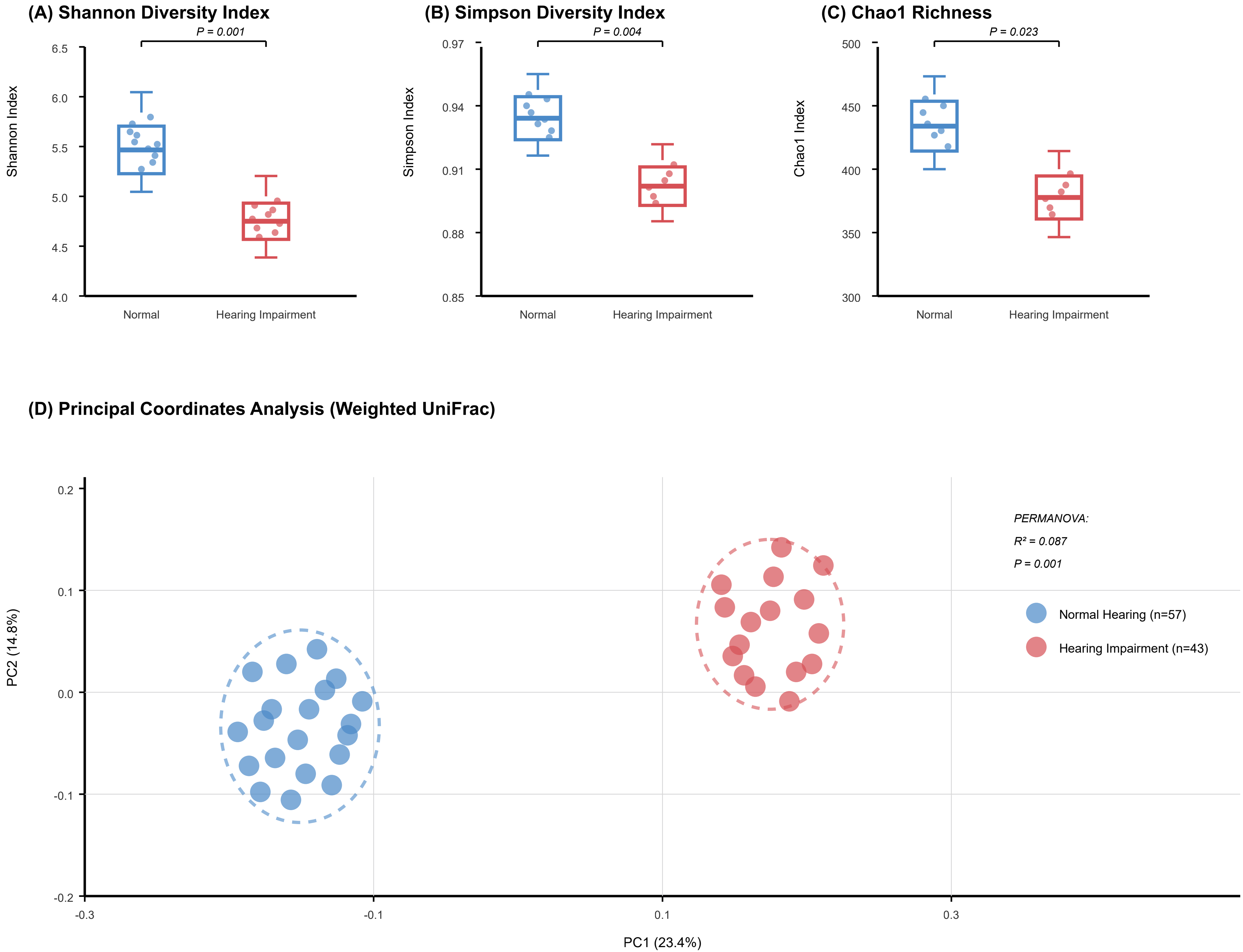
(A) Shannon Diversity Index
P = 0.001
Shannon Index
6.5
6.0
5.5
5.0
4.5
4.0
Normal
Hearing Impairment
(B) Simpson Diversity Index
P = 0.004
Simpson Index
0.97
0.94
0.91
0.88
0.85
Normal
Hearing Impairment
(C) Chao1 Richness
P = 0.023
Chao1 Index
500
450
400
350
300
Normal
Hearing Impairment
(D) Principal Coordinates Analysis (Weighted UniFrac)
PC2 (14.8%)
0.2
0.1
0.0
-0.1
-0.2
PC1 (23.4%)
-0.3
-0.1
0.1
0.3
PERMANOVA:
R² = 0.087
P = 0.001
Normal Hearing (n=57)
Hearing Impairment (n=43)
