# Supplementary material for: The gut-inflammation-hearing axis in diabetic patients aged 30–55: exploring microbiome-mediated pathways to auditory complications
Source: BMC Microbiol. 2026 Aug 15;26:995. doi: 10.1186/s12866-026-05504-8 (PMC13753257; doi:10.1186/s12866-026-05504-8)
Supplement: Supplementary file 3 [file 12866_2026_5504_MOESM3_ESM.pdf]

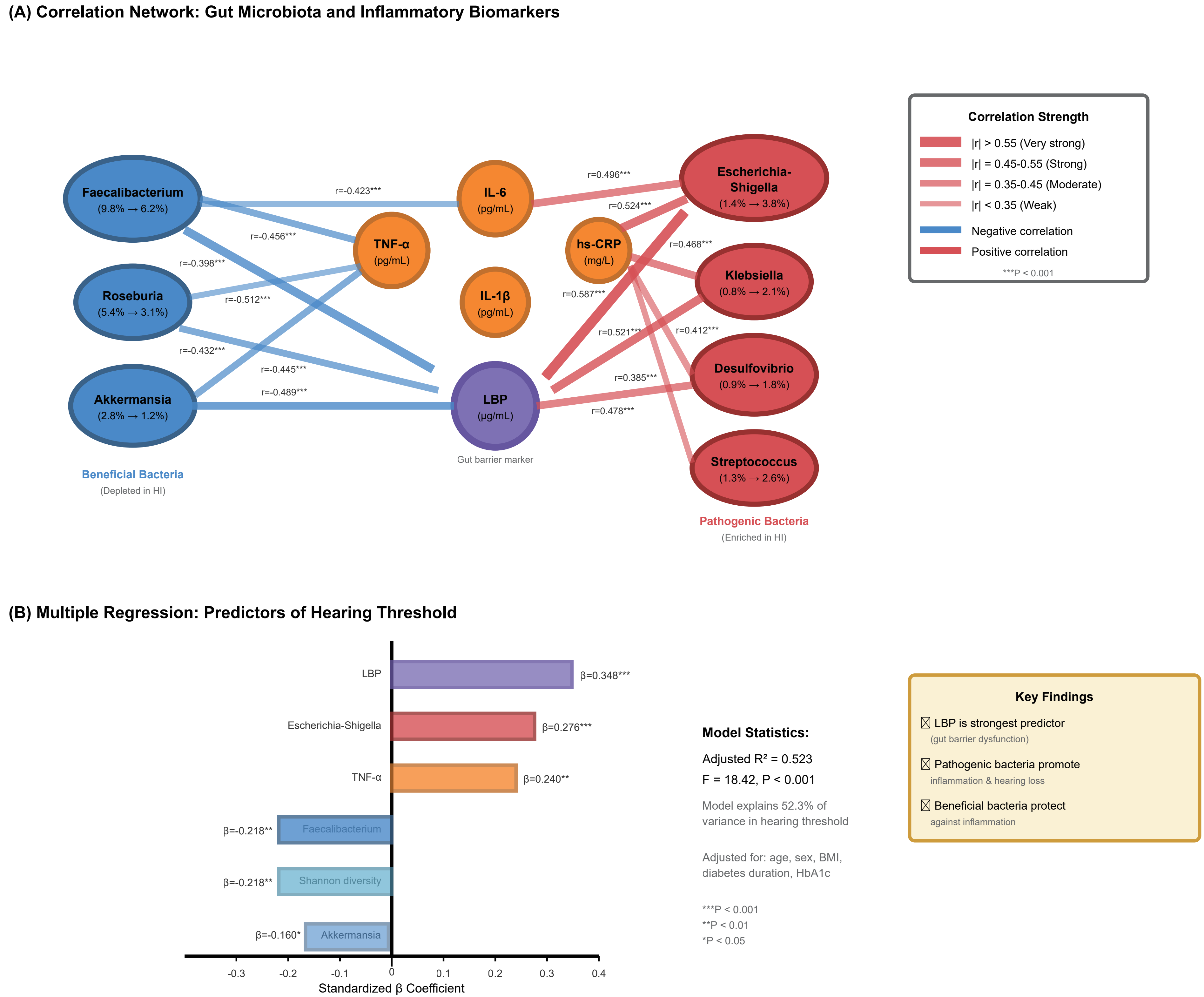
(A) Correlation Network: Gut Microbiota and Inflammatory Biomarkers
Faecalibacterium (9.8% → 6.2%)
Roseburia (5.4% → 3.1%)
Akkermansia (2.8% → 1.2%)
Beneficial Bacteria
(Depleted in HI)
TNF-α (pg/mL)
IL-6 (pg/mL)
IL-1β (pg/mL)
hs-CRP (mg/L)
LBP (μg/mL)
Gut barrier marker
Escherichia-Shigella (1.4% → 3.8%)
Klebsiella (0.8% → 2.1%)
Desulfovibrio (0.9% → 1.8%)
Streptococcus (1.3% → 2.6%)
Pathogenic Bacteria
(Enriched in HI)
r=-0.423***
r=-0.456***
r=-0.398***
r=-0.512***
r=-0.432***
r=-0.445***
r=-0.489***
r=0.496***
r=0.524***
r=0.468***
r=0.587***
r=0.521***
r=0.412***
r=0.385***
r=0.478***
Correlation Strength
|r| > 0.55 (Very strong)
|r| = 0.45-0.55 (Strong)
|r| = 0.35-0.45 (Moderate)
|r| < 0.35 (Weak)
Negative correlation
Positive correlation
***P < 0.001
(B) Multiple Regression: Predictors of Hearing Threshold
LBP β=0.348***
Escherichia-Shigella β=0.276***
TNF-α β=0.240**
Faecalibacterium β=-0.218**
Shannon diversity β=-0.218**
Akkermansia β=-0.160*
-0.3 -0.2 -0.1 0 0.1 0.2 0.3 0.4
Standardized β Coefficient
Model Statistics:
Adjusted R² = 0.523
F = 18.42, P < 0.001
Model explains 52.3% of variance in hearing threshold
Adjusted for: age, sex, BMI, diabetes duration, HbA1c
***P < 0.001
**P < 0.01
*P < 0.05
Key Findings
☒ LBP is strongest predictor
(gut barrier dysfunction)
☒ Pathogenic bacteria promote
inflammation & hearing loss
☒ Beneficial bacteria protect
against inflammation
