# Supplementary material for: The gut-inflammation-hearing axis in diabetic patients aged 30–55: exploring microbiome-mediated pathways to auditory complications
Source: BMC Microbiol. 2026 Aug 15;26:995. doi: 10.1186/s12866-026-05504-8 (PMC13753257; doi:10.1186/s12866-026-05504-8)
Supplement: Supplementary file 4 [file 12866_2026_5504_MOESM4_ESM.pdf]

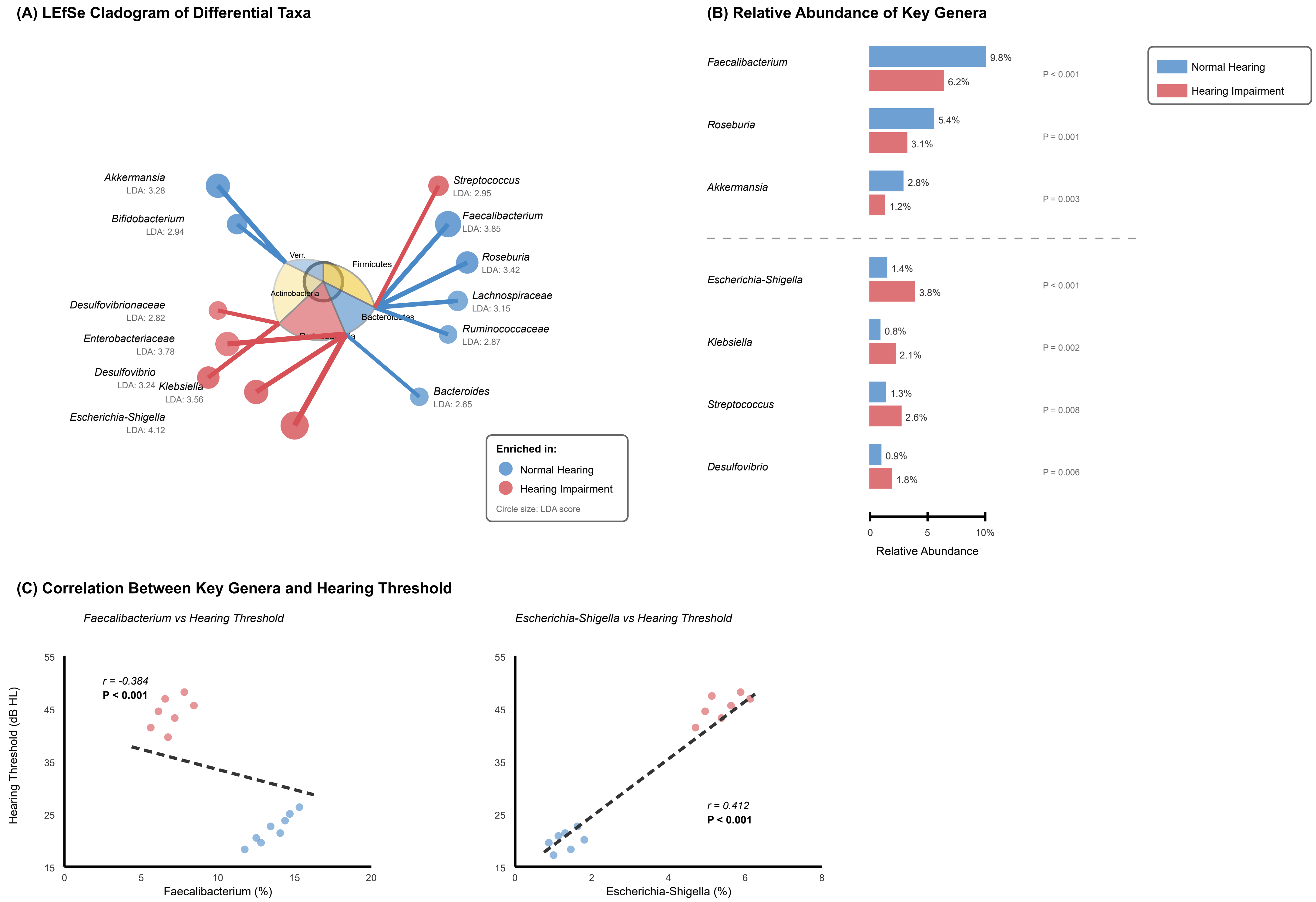

(A) LEfSe Cladogram of Differential Taxa
Akkermansia
LDA: 3.28
Bifidobacterium
LDA: 2.94
Verr.
Firmicutes
Actinobacteria
Streptococcus
LDA: 2.95
Faecalibacterium
LDA: 3.85
Roseburia
LDA: 3.42
Lachnospiraceae
LDA: 3.15
Ruminococcaceae
LDA: 2.87
Bacteroides
LDA: 2.65
Desulfovibrionaceae
LDA: 2.82
Enterobacteriaceae
LDA: 3.78
Desulfovibrio
LDA: 3.24
Klebsiella
LDA: 3.56
Escherichia-Shigella
LDA: 4.12
Enriched in:
Normal Hearing
Hearing Impairment
Circle size: LDA score
(B) Relative Abundance of Key Genera
Normal Hearing
Hearing Impairment
Faecalibacterium
9.8%
6.2%
P < 0.001
Roseburia
5.4%
3.1%
P = 0.001
Akkermansia
2.8%
1.2%
P = 0.003
Escherichia-Shigella
1.4%
3.8%
P < 0.001
Klebsiella
0.8%
2.1%
P = 0.002
Streptococcus
1.3%
2.6%
P = 0.008
Desulfovibrio
0.9%
1.8%
P = 0.006
0
5
10%
Relative Abundance
(C) Correlation Between Key Genera and Hearing Threshold
Faecalibacterium vs Hearing Threshold
r = -0.384
P < 0.001
Hearing Threshold (dB HL)
55
45
35
25
15
0
5
10
15
20
Faecalibacterium (%)
Escherichia-Shigella vs Hearing Threshold
r = 0.412
P < 0.001
55
45
35
25
15
0
2
4
6
8
Escherichia-Shigella (%)
