# Supplementary material for: The gut-inflammation-hearing axis in diabetic patients aged 30–55: exploring microbiome-mediated pathways to auditory complications
Source: BMC Microbiol. 2026 Aug 15;26:995. doi: 10.1186/s12866-026-05504-8 (PMC13753257; doi:10.1186/s12866-026-05504-8)
Supplement: Supplementary file 5 [file 12866_2026_5504_MOESM5_ESM.pdf]

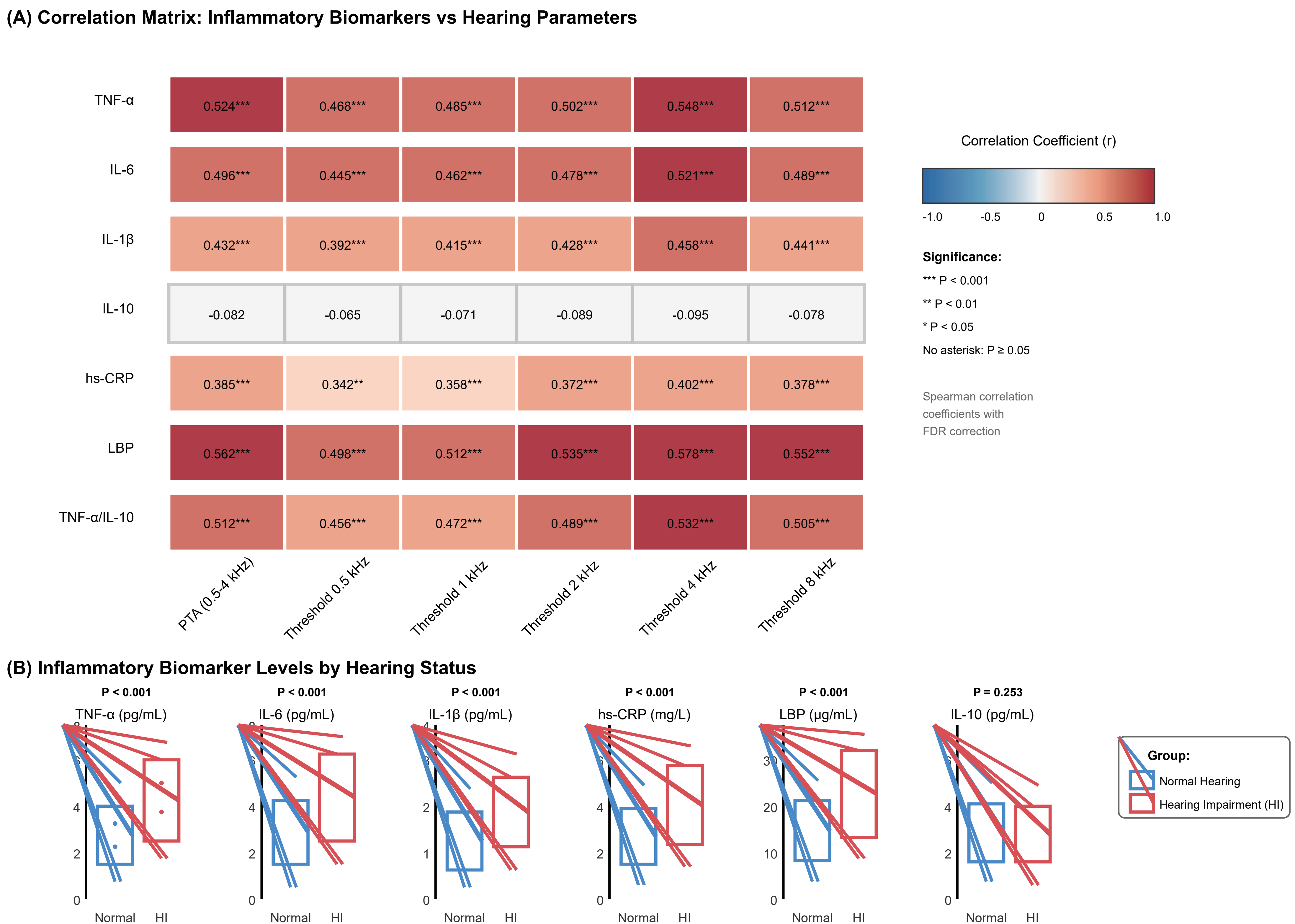

(A) Correlation Matrix: Inflammatory Biomarkers vs Hearing Parameters
TNF-α
0.524*** 0.468*** 0.485*** 0.502*** 0.548*** 0.512***
IL-6
0.496*** 0.445*** 0.462*** 0.478*** 0.521*** 0.489***
IL-1β
0.432*** 0.392*** 0.415*** 0.428*** 0.458*** 0.441***
IL-10
-0.082 -0.065 -0.071 -0.089 -0.095 -0.078
hs-CRP
0.385*** 0.342** 0.358*** 0.372*** 0.402*** 0.378***
LBP
0.562*** 0.498*** 0.512*** 0.535*** 0.578*** 0.552***
TNF-α/IL-10
0.512*** 0.456*** 0.472*** 0.489*** 0.532*** 0.505***
PTA (0.5-4 kHz)
Threshold 0.5 kHz
Threshold 1 kHz
Threshold 2 kHz
Threshold 4 kHz
Threshold 8 kHz
Correlation Coefficient (r)
-1.0
-0.5
0
0.5
1.0
Significance:
*** P < 0.001
** P < 0.01
* P < 0.05
No asterisk: P ≥ 0.05
Spearman correlation
coefficients with
FDR correction
(B) Inflammatory Biomarker Levels by Hearing Status
P < 0.001
TNF-α (pg/mL)
P < 0.001
IL-6 (pg/mL)
P < 0.001
IL-1β (pg/mL)
P < 0.001
hs-CRP (mg/L)
P < 0.001
LBP (μg/mL)
P = 0.253
IL-10 (pg/mL)
Normal
HI
Group:
Normal Hearing
Hearing Impairment (HI)
