# Supplementary material for: The gut-inflammation-hearing axis in diabetic patients aged 30–55: exploring microbiome-mediated pathways to auditory complications
Source: BMC Microbiol. 2026 Aug 15;26:995. doi: 10.1186/s12866-026-05504-8 (PMC13753257; doi:10.1186/s12866-026-05504-8)
Supplement: Supplementary file 6 [file 12866_2026_5504_MOESM6_ESM.pdf]

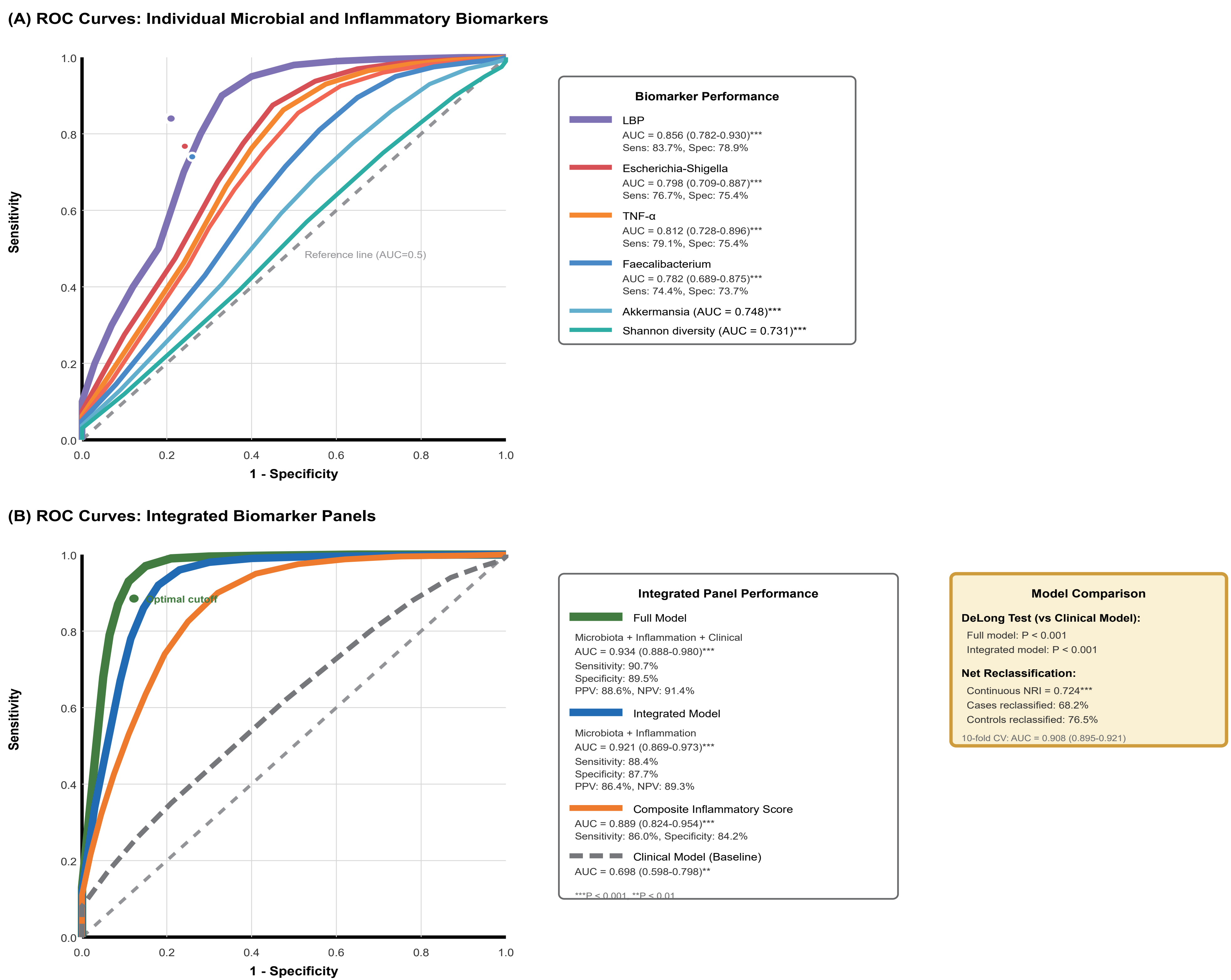
(A) ROC Curves: Individual Microbial and Inflammatory Biomarkers
Sensitivity
1 - Specificity
Reference line (AUC=0.5)
Biomarker Performance
LBP
AUC = 0.856 (0.782-0.930)***
Sens: 83.7%, Spec: 78.9%
Escherichia-Shigella
AUC = 0.798 (0.709-0.887)***
Sens: 76.7%, Spec: 75.4%
TNF-α
AUC = 0.812 (0.728-0.896)***
Sens: 79.1%, Spec: 75.4%
Faecalibacterium
AUC = 0.782 (0.689-0.875)***
Sens: 74.4%, Spec: 73.7%
Akkermansia (AUC = 0.748)***
Shannon diversity (AUC = 0.731)***
(B) ROC Curves: Integrated Biomarker Panels
Sensitivity
1 - Specificity
Optimal cutoff
Integrated Panel Performance
Full Model
Microbiota + Inflammation + Clinical
AUC = 0.934 (0.888-0.980)***
Sensitivity: 90.7%
Specificity: 89.5%
PPV: 88.6%, NPV: 91.4%
Integrated Model
Microbiota + Inflammation
AUC = 0.921 (0.869-0.973)***
Sensitivity: 88.4%
Specificity: 87.7%
PPV: 86.4%, NPV: 89.3%
Composite Inflammatory Score
AUC = 0.889 (0.824-0.954)***
Sensitivity: 86.0%, Specificity: 84.2%
Clinical Model (Baseline)
AUC = 0.698 (0.598-0.798)**
***P < 0.001, **P < 0.01
Model Comparison
DeLong Test (vs Clinical Model):
Full model: P < 0.001
Integrated model: P < 0.001
Net Reclassification:
Continuous NRI = 0.724***
Cases reclassified: 68.2%
Controls reclassified: 76.5%
10-fold CV: AUC = 0.908 (0.895-0.921)
